# Efficacy of administered mesenchymal stem cells in the initiation and co‐ordination of repair processes by resident disc cells in an ovine (Ovis aries) large destabilizing lesion model of experimental disc degeneration

**DOI:** 10.1002/jsp2.1037

**Published:** 2018-10-10

**Authors:** Cindy C. Shu, Andrew Dart, Robin Bell, Christina Dart, Elizabeth Clarke, Margaret M. Smith, Christopher B. Little, James Melrose

**Affiliations:** ^1^ Raymond Purves Bone and Joint Research Laboratory, Kolling Institute, Northern Sydney Local Health District St. Leonards New South Wales Australia; ^2^ Faculty of Medicine and Health University of Sydney, Royal North Shore Hospital St. Leonards New South Wales Australia; ^3^ University of SydneyVeterinary Teaching Hospital Camden New South Wales Australia; ^4^ Murray Maxwell Biomechanics Laboratory, Kolling Institute of Medical Research, The Royal North Shore Hospital University of Sydney St Leonards New South Wales Australia; ^5^ Sydney Medical School, Northern The University of Sydney St Leonards New South Wales Australia; ^6^ Graduate School of Biomedical Engineering University of New South Wales Sydney New South Wales Australia

**Keywords:** intervertebral disc, intervertebral disc degeneration, intervertebral disc repair, mesenchymal stem cells

## Abstract

**Background:**

Forty percent of low back pain cases are due to intervertebral disc degeneration (IVDD), with mesenchymal stem cells (MSCs) a reported treatment. We utilized an ovine IVDD model and intradiscal heterologous MSCs to determine therapeutic efficacy at different stages of IVDD.

**Methodology:**

Three nonoperated control (NOC) sheep were used for MSC isolation. In 36 sheep, 6 × 20 mm annular lesions were made at three spinal levels using customized blades/scalpel handles, and IVDD was allowed to develop for 4 weeks in the Early (EA) and late Acute (LA) groups, or 12 weeks in the chronic (EST) group. Lesion IVDs received injections of 10 × 10^6^ MSCs or PBS, and after 8 (EA), 22 (LA) or 14 (EST) weeks recuperation the sheep were sacrificed. Longitudinal lateral radiographs were used to determine disc heights. IVD glycosaminoglycan (GAG) and hydroxyproline contents were quantified using established methods. An Instron materials testing machine and customized jigs analyzed IVD (range of motion, neutral zone [NZ] and stiffness) in flexion/extension, lateral bending and axial rotation. qRTPCR gene profiles of key anabolic and catabolic matrix molecules were undertaken. Toluidine blue and hematoxylin and eosin stained IVD sections were histopathologically scoring by two blinded observers.

**Results:**

IVDD significantly reduced disc heights. MSC treatment restored 95% to 100% of disc height, maximally improved NZ and stiffness in flexion/extension and lateral bending in the EST group, restoring GAG levels. With IVDD qRTPCR demonstrated elevated catabolic gene expression (*MMP2/3/9/13, ADAMTS4/5*) in the PBS IVDs and expession normalization in MSC‐treated IVDs. Histopathology degeneracy scores were close to levels of NOC IVDs in MSC IVDs but IVDD developed in PBS injected IVDs.

**Discussion:**

Administered MSCs produced recovery in degenerate IVDs, restored disc height, composition, biomechanical properties, down regulated MMPs and fibrosis, strongly supporting the efficacy of MSCs for disc repair.

## 1 INTRODUCTION

The IVD is a fibrocartilaginous visco‐elastic weight bearing cushion that provides mechanical stability and spinal flexibility during axial loading, flexion and rotation.^1^, ^2^, ^3^ The IVD is composed of superior and inferior cartilagenous end plates (CEPs) which interface with the vertebrae, the outer region of the disc, the annulus fibrosus (AF) is a collagen rich tissue which provides mechanical strength during stretching and in tension.^4^ The AF and the CEPs encompass the central nucleus pulposus (NP), a proteoglycan‐rich tissue which provides the IVD with it's ability to withstand compressive loading. With aging, aggrecan the major IVD proteoglycan,^5^ is susceptible to proteolytic degradation^6^, ^7^ resulting in a reduction in NP water‐imbibing capacity, loss of hydrodynamic weight‐bearing properties,^8^ compromised IVD biomechanical competence, loss of NP proteoglycans,^9^ diminished disc height and the appearance of clefts and fissures in the AF, symptomatic of a diminished IVD capacity to act as a weight bearing structure.^10^

A 10‐year global study of 291 major human diseases placed LBP as the number one musculoskeletal disorder in terms of years lived with disability, an estimated 80% of the general population are affected by LBP, its incidence peaks in the fifth and sixth decade.^11^ Other studies have emphasized the socioeconomic impact of degenerative disc disease (DDD) and LBP. UK costings for LBP of £12.3 billion,^12^ and $9.17 billion for Australia have been published.^13^ The American Academy of Pain Medicine published annual costs in 2006 for chronic pain of $560 to 635 billion, with 53% of all chronic pain patients in the USA affected by LBP, 31 million people have LBP at any one time.^14^ In 2015, the global point prevalence of activity‐limiting LBP of 7·3% indicated that 540 million people were affected globally by LBP at any one time, and today LBP is now recognized as the number one musckuloskeletal condition and cause of disability world‐wide.^11^, ^15^ In 1999, the World Health Organization (WHO) published the IRIS low back pain initiative^16^ designating LBP and stem cell research to restore functional IVDs as high priority research areas. LBP was made a national priority area by the National Health and Medical Research Council (NHMRC) in 2009.^17^

Since their discovery in the late 1960s mesenchymal stem cells (MSCs) have been the subject of intense investigation due to their remarkable efficacy in tissue repair. MSCs were originally considered to migrate into sites of injury, where they engrafted, and differentiated into functional cells, resulting in regeneration of damaged or diseased connective tissue.^18^ Results over the past few decades from several hundred animal studies and many human clinical trials have challenged this mode of action. There is no doubt that MSCs exhibit a remarkable ability to repair diseased tissues, but it has become increasingly apparent that they do not engraft in enough numbers or for sufficient durations in tissue defects to explain the tissue repair and clinical benefit they provide. A recent positron emission tomography (PET) imaging study showed that MSCs remained viable for 3 weeks when injected into canine IVDs in an annular lesion model of disc degeneration^19^ yet beneficial effects with regards to matrix repair and alleviation of LBP were measurable for up to 6 months post operatively (PO). Further modes of action for MSCs in the healing process have therefore been proposed based on the ability of MSCs to enhance resident cell viability and/or proliferation, reduce cell apoptosis,^20^, ^21^ and, in some cases, modulate immune responses.^22^, ^23^, ^24^, ^25^, ^26^ These are due to paracrine effects due to secreted growth factors, cytokines, and hormones from the MSCs and cell‐cell interactions mediated through communicating nanotubes, which convey extracellular vesicles containing reparative peptides/proteins, mRNA, and microRNAs.^18^ Caplan proposed that stem cells should be renamed *Medicinal Signaling Cells* to more accurately reflect how they home in on injured or diseased tissue sites secreting bioactive factors with immunomodulatory and trophic properties which direct the resident cells to undertake the tissue repair process, this may happen long after the MSCs were present in the defect site.^27^ The use of MSCs to undertake IVD repair/regeneration is an area which shows much promise^28^, ^29^, ^30^ with many publications appearing in the literature dealing with animal model based laboratory studies,^31^, ^32^, ^33^, ^34^, ^35^, ^36^ preclinical studies,^37^, ^38^ clinical trials^39^ (Tables 1 and 2) and reviews advocating the use of MSCs for disc repair/regeneration.^28^, ^29^, ^45^, ^46^, ^47^, ^48^, ^49^, ^50^, ^51^

**Table 1:** Use of MSCs and other therapeutic progenitor cells for the treatment of disc degeneration and alleviation of low Back pain

Study	Cell type	Number of cells administered/disc	Reference
Orozco et al^37^	Autologous bone marrow MSCs	10 ± 5 × 10^6^	37
Coric et al^40^	Allogeneic juvenile articular chondrocytes	1–2 × 10^7^	40
Pettine et al^41^	Autologous bone marrow concentrate	121 ± 11 × 10^6^	41
Mochida et al^42^	Autologous reactivated NP cells	1 × 10^6^	42
Elabd et al^43^	Autologous bone marrow MSCs	31 ± 14 × 10^6^	43

Data modified from.^44^

**Table 2:** Clinical trials for treatment of disc degeneration and alleviation of low back pain

Sponsor, start‐end date country	Cell type and number used	Government ID	Study title, database information and when accessed
*A. Use of therapeutic allogeneic and autologous stem cell preparations*			
Red de Terapia Celular 2010‐unspecified Spain	Autologous bone marrow MSCs 0.5‐1.5 × 10^6^ /kg body weight	NCT01513694	*Clinical trial based on the use of mesenchymal stem cells from autologous bone marrow in patients with lumbar intervertebral degenerative disc disease* US National Library of MedicinedClinicalTrials.gov 2012 https://clinicaltrials.gov/ct2/show/study/ NCT01513694.(accessed 20th July 2018)
Mesoblast Ltd 2011‐2015 USA‐Australia	Allogeneic mesenchymal precursor cells 6‐18 × 10^6^	NCT01290367	*Safety and preliminary efficacy study of mesenchymal precursor cells (MPCs) in subjects with lumbar Back pain* US National Library of MedicinedClinicalTrials.gov 2011 https://clinicaltrials.gov/ct2/show/NCT01290367.(accessed 20th July 2018)
Biostar 2012‐2014 South Korea	Autologous adipose derived MSCs 4 × 10^7^	NCT01643681	*Autologous adipose tissue derived mesenchymal stem cells transplantation in patient with lumbar intervertebral disc degeneration* US National Library of MedicinedClinicalTrials.gov 2012 https://clinicaltrials.gov/ct2/show/record/ NCT01643681. (accessed 20th July 2018)
Red de Terapia Celular 2013‐2016 Spain	Allogeneic bone marrow MSCs 10 ± 5 × 10^6^	NCT01860417	*Treatment of degenerative disc disease with allogenic mesenchymal stem cells (MSV) (Disc_allo)* US National Library of MedicinedClinicalTrials.gov 2013 https://clinicaltrials.gov/ct2/show/NCT01860417 .(accessed 20th July 2018)
Inbo Han 2015‐2017 South Korea	Autologous Adipose derived stem cells 2 or 4 × 10^7^	NCT02338271	*Autologous adipose derived stem cell therapy for intervertebral disc degeneration* US National Library of MedicinedClinicalTrials.gov 2015 https://clinicaltrials.gov/ct2/show/NCT02338271 [2016].
Mesoblast Ltd 2015‐2020 USA‐Australia	Allogeneic mesenchymal precursor cells 6 × 10^6^	NCT02412735	*Placebo‐controlled study to evaluate Rexlemestrocel‐L alone or combined with hyaluronic acid in subjects with chronic low Back pain (MSB‐DR003)* US National Library of MedicinedClinicalTrials.gov 2015 https://clinicaltrials.gov/ct2/show/NCT02412735 .(accessed 20th July 2018)
The Foundation for Spinal Research, education and humanitarian care Inc. 2013‐2018 USA	Autologous or allogeneic bone marrow mesenchymal stem cells Cell number used not specified	NCT02529566	*Human autograft mesenchymal stem cell mediated stabilization of the degenerative lumbar spine* US National Library of MedicinedClinicalTrials.gov 2015 available at: https://clinicaltrials.gov/ct2/show/NCT02529566.(accessed 20th July 2018)
Arhus university hospital. 2013‐unspecified Denmark	Autologous bone marrow mesenchymal stem cells Cell number used not specified	EudraCT 2012–003160‐44/DK	*Treatment of moderate intervertebral disc degeneration with autologous bonemarrow derived mesenchymal stem cells (bMSC): EU clinical trial* register‐clinicaltrialregister.eu *, 2013 available at* htpps://www.clinicaltrialsregister.eu/ctr‐search/trial/2012‐003160‐44/DK *(accessed 20th July 2018.*
BioHeart Inc. 2014‐2017 USA	Autologous adipose derived stem cells Cell number used not specified	NCT02097862	*Adipose cells for degenerative disc disease.* US National Library of MedicinedClinicalTrials.gov 2015 available at: https://clinicaltrials.gov/ct2/show/NCT02097862.(accessed 20th July 2018)
*B. Therapeutic articular chondrocytes and disc cells*			
ISTO technologies Inc 2012–2016 USA	Allogeneic juvenile chondrocytes Cell number used not specified	NCT01771471	*A study comparing the safety and effectiveness of cartilage cell injected into the lumbar disc as compared to a placebo* US National Library of MedicinedClinicalTrials.gov 2013 https://clinicaltrials.gov/ct2/show/ NCT01771471 (accessed 20th July 2018).
Tetec Inc 2012‐2021 Austria/Germany	Autologous disc cells Cell number used not specified	EudraCT 2010–023830‐22/AT NCT01640457	*NDisc study: A prospective randomized multicentre phase I / II clinical trial to evaluate safety and efficacy of NOVOCART disc plus autologous disc chondrocyte transplantation (ADCT) in the treatment of Nucleotomized and degenerative lumbar discs to avoid secondary disease.* EU clinical trial register‐clinicaltrialregister.EU, 2012 available at htpps://www.clinicaltrialsregister.eu/ctr‐search/trial/2010‐023830‐22/AT (accessed 20th July 2018). *Safety and efficacy with NOVOCART disc plus (ADCT) for the treatment of degenerative disc disease in lumbar spine (NDisc)* US National Library of MedicinedClinicalTrials.gov 2015 available at: https://clinicaltrials.gov/ct2/show/NCT01640457 (accessed 20th July 2018)

Data modified from.^44^

Most pre‐clinical studies have treated IVDs with MSCs acutely after induction of disc degeneration, and no direct studies on the relative efficacy of MSCs in different stages of the disease process have been evaluated. This is important given the clinical presentation of patients once pathology is well established and asks the question can MSCs be used to “treat” rather than “prevent” IVDD? While clinical trials have and are being undertaken (Table 2), the data from the current study will better inform prospective trial design and provide insights as to how patient selection should be made. Based on the findings of the present study there is much to be looked forward to in the therapeutic application of MSCs for the treatment of DDD/LBP.

The aim of the present study was to assess the efficacy of ovine bone marrow stromal stem cells for repair of the degenerate IVD. We used an aggressive ovine model where disc degeneration was induced by a large controlled 6 mm deep by 20 mm wide surgical annular lesion. A wide range of methodologies were used to assess the IVD repair process including testing of the material properties of IVDs using customized in‐house developed jigs in an Instron servo‐hydraulic materials testing machine. Pre‐ and Post‐Surgery lateral X‐rays were used to calculate disc heights in each of the treatment groups. Biochemical tissue compositional analyses and catabolic and anabolic matrix gene profiling were also used to characterize the degenerative changes in IVDD, and in IVD repair tissue induced by MSC treatment. The histopathology of these IVDs was systematically scored using a recently developed, validated histopathology scoring scheme.^52^ A distinguishing feature of this study besides its multidisciplinary evaluations of IVD degeneration and repair processes by MSCs, is the use of a large 6 mm deep and 20 mm wide annular lesion to induce IVD degeneration. This is the only study to ever use such a large defect thus the positive repair responses we observed with MSCs are particularly significant noteworthy findings. Furthermore, in any prospective clinical therapeutic application it is important to select patients with appropriate disease duration and symptoms which can respond effectively to the treatment being developed. In our study, IVD degeneration was induced for a relatively short time period of 1 month and relatively short and longer recuperative periods of 8 or 22 weeks respectively used to ascertain if spontaneous repair occurred in these time periods. These simulate early acute (EA group) and late acute (LA group) stages of the disease process. We also established IVD degeneration over a 3 month period and then allowed a modest recuperative period of 14 weeks in a third group of sheep (EST group). This group simulated the chronic phases of IVD degeneration. Thus we were able to evaluate the efficacy of MSCs for IVD repair in these three treatment groups which simulated early and late acute, and established chronic stages of IVD degeneration. Our positive findings with administered MSCs points to the efficacy of this procedure in the treatment of all three phases of IVD degeneration.

## 2 METHODS

### 2.1 Chemicals and consumables

All chemical and supplier details are as specified earlier^53^, ^54^ except where noted.

### 2.2 Animal welfare and ethics

A total of 51 2 to 3‐year‐old merino wethers (castrated males) were purchased from local sale yards for this study and held in open paddocks till required. All animal welfare and ethics for this work were approved by the University of Sydney Animal Care and Ethics Committee under ethics approval A45/6‐2011/3/5544.

### 2.3 Sheep acclamitization following purchase and recovery details post surgery

Following sheep purchase the sheep were acclamitized as a single flock and allowed to roam freely in an open paddock for 3 weeks. Sheep were then randomly divided into Nonoperated‐control (NOC) and three IVDD cohorts (Early Acute (EA), Late Acute (LA) and Established (EST) each of 12 sheep held in holding pens adjacent to the operating suite for 1 week. Surgery (detailed below) was conducted on the 12 sheep in each group in 1 day. Following surgery, the sheep were housed in recovery pens for 1 to 2 hours post surgery before being transferred back to the main holding pens for 1 week recovery, at which stage they were ready to be transferred to an open paddock and managed as a single flock until required for the second surgery. At the specified time post IVDD induction half of the sheep in the EA, LA and EST treatment groups received MSC injections and the remainder PBS carrier in each of their three injured discs. Following recovery from the second surgery sheep were again penned for 1 week then transferred to an open paddock as a single flock with NOCs until sacrifice. Feeding of the sheep during their residency in pens was with lucerne or oat/vetch hays supplemented with sheep pellets, roughage and water ad‐libitum. In the open paddock the sheep were free to graze on pasture grasses, sheep pellets and lucerne hay were also made available.

### 2.4 Demonstration of MSC authenticity and multipotency

The methodology employed to demonstrate MSC authenticity and multipotency were as recommended by The International Society For Cellular Therapy namely (a) MSCs expressed CD105, CD73, CD44 and CD90, and not CD45, CD34, CD31, CD14 CD19 and HLA‐DR surface molecule (Figure S1, Supporting Information); (b) MSCs were plastic‐adherent when maintained in standard culture (c) and had the ability to differentiate into adipocytes, osteoblasts and chondrocytes in vitro (details are provided below).

### 2.5 Isolation of mesenchymal stem cells

Pooled bone marrow aspirate from the iliac crest of 3 NOC donor sheep in Na_2_EDTA/Tris‐buffered saline, pH 7.2, was gently mixed by inversion and centrifuged at 3000 rpm for 20 minutes in a swing‐out rotor. The buffy coat cells at the interface were collected, washed twice in sterile PBS and allowed to attach overnight to tissue culture flasks in DMEM supplemented with 10% FBS (AusGeneX, Molendinar, QLD Australia), antibiotics (Penicillin/streptomycin) and 2 mM L‐glutamine. Nonadherent cells were washed away and the cells cultured till confluent, detached with typsin/EDTA and re‐passaged at a density of 1.5 × 10^6^ cells/T75 flask or 5 × 10^6^ cells/T175 flask. MSCs from passage 3 were cryopreserved at 5 × 10^6^ MSCs/cryovial in 0.5 mL DMEM +20% FCS + 10% v/v DMSO. To prepare MSCs for injection, 5 × 10^6^ cells were seeded into T175 flasks and expanded over several passages until sufficient cell numbers had been achieved (population doubling level, PDL = 11).

### 2.6 Demonstration of the multipotency of the mesenchymal stem cell preparation

The actual MSC preparations used on each day for the sheep treatments were tested to ensure that they retained tri‐lineage differentiation capacity consistently with other MSC preparations.

*Chondrogenesis*: 250 000 MSCs were pelleted by centrifugation at 500 g in chondrogenic selection media (ChondroDiff, Miltenyi Biotec, Mcquarie Park, NSW Australia) and cultured for 21 days with media changes every 2 to 3 days; cell pellets were washed in PBS, fixed in 10% neutral buffered formalin for 4 hours and stored in 70% ethanol, then dehydrated in sequential ethanol solutions and xylene then embedded in paraffin. Microtome sections (4 μm) were stained with toluidine blue‐fast green to visualize tissue proteoglycans.

*Osteogenesis*: 10 000 MSCs/well seeded in 24 well plates were cultured for 10 days in osteogenic selection media (OsteoDiff, Miltenyi Biotec), which was changed every 3 to 4 days; calcium deposition was detected by Alizarin Red S staining of monolayers using 0.37% w/v Alizarin red S, pH 4.2.

*Adipogenesis*: 50000 MSCs/well seeded in 12 well plates were cultured in αMEM containing 15% FCS, 10 mM L‐glutamine, 0.5 μM dexamethasone, 0.5 μM IBMS and 50 μM indomethacin for 2 weeks, media was changed every 3 to 4 days; Adipogenesis was demonstrated by triglyceride staining on formalin fixed monolayers using Oil Red O (5 g/mL in 70% v/v isopropanol) at 37**°**C.

### 2.7 Induction of disc degeneration and administration of MSCs

#### 2.7.1 The annular incision model

The surgical model used to induce disc degeneration was as described earlier.^53^ Pre‐surgical sedation was provided using intravenous (IV) diazepam (0.2 mg/kg) and ketamine (5 mg/kg), pre‐operative analgesia was administered 12 hours prior to surgery using a slow release fentanyl patch (75 mg) and with intramuscular (IM) injection of Carprofen (5 mg/kg IV). Lumbosacral xylazine (0.05 mg/kg in 1 mL sterile saline) was administered to provide analgesia and muscle relaxation at the end of the lesion induction surgery. Animals received prophylactic antibiotics/opioid to reduce infection and minimize pain (Ceftiofur 5 mg/kg IV‐1 dose pre‐ and 1 dose PO) and methadone (0.1 mg/kg IV prior to surgery and 0.15 mg/kg IM PO). Buprenorphine (0.005 mg/kg IM) was also administered once during PO recovery.

General anesthesia was induced by mask and maintained by endotracheal intubation of 1.5% to 5% halothane in a 33% nitrous oxide 66% oxygen mixture at a flow rate of 2 and 4 L/min respectively. Spinal surgery was performed using standard aseptic technique, hemostasis was maintained using electrocautery. The wool was shorn from the surgical operative site and this was scrubbed with sequential povidone iodine and alcohol scrubs. A skin incision (~20 cm long) was made from the last rib to the pelvis. The fascia was incised and psoas muscles retracted ventrally to expose IVDs avoiding entering into the peritoneal cavity. The controlled 6 mm deep and 20 mm wide anterolateral annular incisions in L1L2, L3L4 and L5L6 IVDs were made using two edge by edge incisions with an Abbott and Mann No 9 scalpel blade and customized scalpel handles which allowed a controlled maximum penetration depth of the lesion to a depth of 6 mm (Figure 3A‐L). This formed a reproducible 6 × 20 mm incision in the AF whose extent was visible in a horizontally bisected IVD by the penetration of blood vessels into the lesion site (Figure 3E). Standard sized 2 to 3 year old merino wethers (67.05 ± 6.5 kg) were selected to ensure that the IVD lesion penetrated only as far as the inner AF.

The surgical wound was closed in 3 layers. The psoas muscle was repositioned by suturing the adjacent fascia, and deep dermis was also sutured with 2/0 ethicon absorbable vicryl and the skin was closed with 0 PDS sutures. Sheep recovery following surgery was carefully monitored. Sheep were initially held in a confined pen in a recumbant position for 30 to 60 minutes after which they were transferred to covered holding pens once they had regained a standing posture and held in these pens in groups of up to 12 sheep for up to 1 week then transferred to an open paddock where they were allowed to roam freely. The animals were carefully monitored throughout this period for any signs of distress or lameness by experienced board certified veterinary personnel.

#### 2.7.2 Injection of MSCs

In a pilot study, chloromethylbenzamido CellTrackerTM CM‐DiI fluorescent dye was used to label MSCs prior to injection.^55^ CM‐DiI is a lipophilic mildly thiol‐reactive chloromethyl fluor that conjugates to thiol‐containing peptides and proteins in cell membranes. Cells are highly permeable to CM‐DiI which is retained in cells throughout aldehyde fixation, permeabilization, and paraffin embedding procedures Injection of MSCs (1 × 10^6^ cells in 0.2 mL PBS) was undertaken with a hypodermic syringe and 23G needle. The length of these needles is 23 mm, injection of MSCs 9 to 10 mm into the central NP through the contralateral AF away from the lesion site achieved administration of MSCs into the IVD and was confirmed by histology using fluorescent and brightfield imaging (Figure 3C,D).

**Figure 1:**
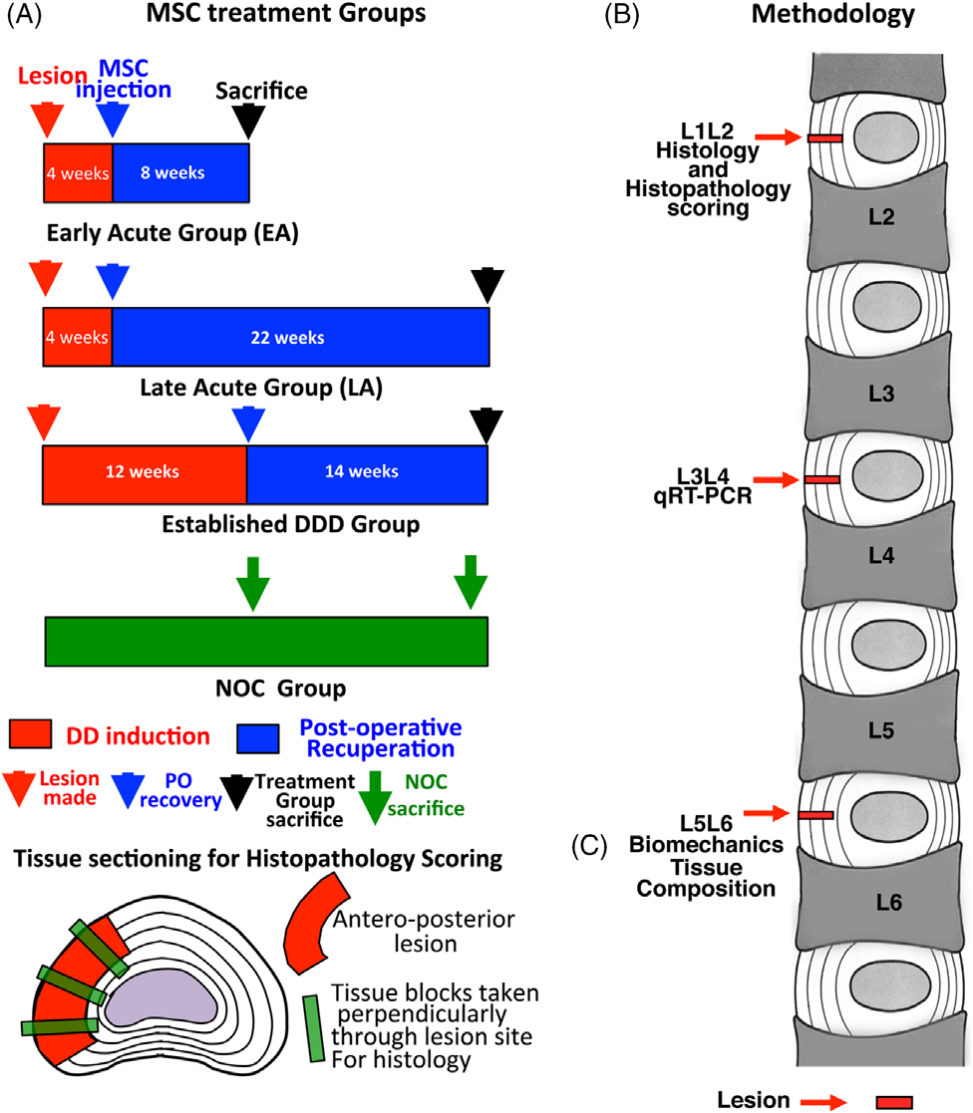
**(**A) Timetable for the lesion induction, MSC injection, recuperation and sacrifice of the three treatment protocols used in this study. (B) Demonstration of the pluripotency of the MSC preparation used in this study. (B) Summary of the analyses used in this study and the spinal levels examined. (C) Schematic of the lesion location and the histological sampling of the lesion site

Once IVD degeneration had been established for 4 or 12 weeks (Figure 1A) and the surgical wound site had healed, the lumbar IVDs were exposed by a second surgery on the contralateral side of the AF away from the original lesion site, and MSCs were injected into the NP (Figure 3C,D). Histology confirmed the MSCs were localized in the NP with a limited number along the injection tract, and minimal leakage of MSCs from the injection site (Figure 3C,D).

Sheep were sacrificed 8, 22 or 14 weeks after MSC treatment [EA, LA, EST groups respectively (see Figure 1A)]. Twelve age and sex matched sheep were used as nonoperated, noninjected controls (NOC), these were sacrificed at times equal to 3 or 6 months after the lesion surgery was conducted. L1L2 IVDs were used for histology, L3L4 for gene expression and L5L6 for biomechanics and biochemical analyses (see Figure 1A,B).

### 2.8 Spinal imaging

Lateral and dorsal longitudinal plain radiographs were taken pre‐ and post‐lesion surgery, pre‐injection, and at the termination of the experiments. Surgical wire was placed through adjacent spinal processes at lesion levels as landmarks on the X‐rays to confirm the location of the lesion IVDs. Landmarks were also placed on scanned images of the X‐rays for calculation of the disc height index (DHI).^56^

### 2.9 Biomechanical studies

The L5L6 IVDs and adjacent vertebral body segments were isolated for spinal biomechanical testing using a custom apparatus attached to an Instron servo‐hydraulic bi‐axial materials testing machine.^57^ The specimens were covered by saline‐soaked gauze to maintain hydration prior to testing. After all posterior bony elements (dorsal lamina, facet joints) were removed, K‐wires were inserted into the vertebral bodies which were then immersed in polymethyl methacrylate for rigid clamping. The functional spinal units were then placed in customized jigs and loaded in flexion‐extension, lateral bending or axial rotation with no axial compression at 5°/sec to a torque limit of ±5 Nm for 10 cycles. Torque (Instron dynacell, 50 Nm capacity) and angle data were collected at 20 Hz using the Instron data acquisation system, and the final full cycle was used for analyses. The range of motion (ROM), neutral zone (NZ) and stiffness were measured from cyclic torque‐angle curves as previously described.^57^ Briefly, ROM was measured as the total angular deflection from maximal positive to minimal negative torque. For NZ and stiffness calculation, first the torque‐angle data were fitted with a seventh order polynomial using an in‐built function in Matlab (Mathworks, Natick, Massachusetts). The neutral zones for each loading direction were calculated as the angular range where the gradient of the torque‐angle curve was <0.05 Nm/deg, then the final neutral zone was the common overlapping range (Figure 6). The initial stiffness was calculated for each loading direction as the gradient of the torque‐angle curve at the neutral position (averaged over the range where the change in gradient was <0.05 Nm/deg). The final stiffness was the average from both loading directions.

### 2.10 Compositional analysis of disc tissues

Following biomechanical testing, the IVDs were bisected, then zonally dissected into AF1, AF2 and NP (Figure 1A), finely diced and digested with papain overnight at 60°C (20 μL papain suspension, 7.9 mg cysteine per 10 mL PBS containing 10 mM EDTA, pH 7.0). Triplicate aliquots of the solubilized tissues were measured for sulfated glycosaminoglycan (GAG) using 1, 9‐dimethylmethylene blue as specified earlier.^58^ Tracheal chondroitin sulfate (Sigma C8529) was used as standard. (Figure 4) Aliquots of the solubilized tissues were dried by SpeedVac, hydrolysed at 110°C overnight in 6 M HCl, neutralized with 6 M NaOH, and hydroxyproline contents determined on triplicate samples using the dimethylaminobenzaldehyde method of Stegemann and Stalder.^59^

### 2.11 Histological processing of IVDs

Prior to fixation, all soft and most bony tissue surrounding the IVD were trimmed from the specimens using scalpels and a Dremel router to trim bone and minimize the decalcification time for the specimens. After en‐bloc fixation for 48 hours in 10% neutral‐buffered formalin the specimens were decalcified over 8 days in 10% formic acid/5% formalin with constant agitation. Decalcification solution was replaced every 48 hours. Vertical tissue blocks (4 mm) were then taken of the IVD‐vertebral bodies perpendicularly through the lesion site (Figure 1C). Tissue blocks were dehydrated in graded ethanol solutions, xylene and equilibrated in methyl benzoate prior to embedding in paraffin wax. Microtome sections (4 μm) were cut from the fixed decalcified tissue blocks and attached to Super Frost Plus glass microscope slides (Menzel‐Glaser, Germany), de‐paraffinized in xylene, and re‐hydrated through graded ethanol to water.

### 2.12 Toluidine blue staining

Decalcified sections of IVD/vertebral bodies were stained in 0.04% w/v toluidine blue in 0.1 M sodium acetate buffer, pH 4.0 for 10 minutes, to visualize GAGs followed by a 2 minutes counterstain in 0.1% w/v fast green FCF to contrast the GAG stained areas (Figure 5).

### 2.13 Hematoxylin and eosin staining

Tissue sections were stained in Harris's Hematoxylin (5 minutes), rinsed in tap water, blued in Scotts Blueing solution (1 minute) and counterstained in eosin (5 minutes), dehydrated in absolute ethanol, cleared in xylene and mounted.

### 2.14 Histopathological scoring of tissue sections

Tissue sections were scored using a quantitative validated scoring system which evaluates toluidine blue and hematoxylin and eosin (H&E) stained tissue sections of degenerate IVDs and those which had been injected with MSCs.^52^ This scheme is based on (a) toluidine blue GAG staining levels, (b) Structural characteristics of the lesion, (c) Cellular morphology in and around the lesion site, (d) Blood vessel ingrowth into the lesion repair site, (e) Cellular infiltration into the lesion site, (f) specific degenerative or repair response features associated with the lesion site such as chondroid metaplasia, cystic degeneration, denudation of collagen fibrillar networks leaving collagen fibers devoid of associated proteoglycan. These criteria were individually scored as outlined in Table S1 to provide a cumulative histopathology score.

### 2.15 Real‐time polymerase chain reaction

The L3L4 IVDs were dissected into AF1, AF2 and NP zones as depicted in Figure 1A and 80‐100 mg portions were snap frozen in liquid nitrogen. Frozen tissues were pulverized using a Mikro Dismembrator (Sartorius AG, Goettingen, Germany). Total RNA was extracted from the tissue powders using Trizol (Life Technologies, Melbourne, VIC Australia) and Qiagen RNA isolation columns (Chadstone, VIC Australia). RNA (1 μg) from each sample was reverse transcribed (GoScript, Promega, Hawthorn East, VIC Australia) using random pentadecamers (50 ng/mL, Sigma‐Genosys, Castle Hill, NSW Australia) and RNase inhibitor (10 U/reaction, Bioline, Alexandria, NSW, Australia). The resultant cDNA was subjected to real‐time polymerase chain reaction (qRT‐PCR) in a Rotogene 6000 (Corbett Life Sciences, Qiagen) using Immomix (Bioline), SYBR Green I (Cambrex Bioscience, Rutherford, NJ) and 0.3 mM primers (Sigma‐Genosys, Table S2). Standard curves were generated using pooled IVD cDNA and relative copy numbers for genes calculated. Sample loadings were normalized on the basis of total RNA content of the samples since we have found that the expression of commonly used housekeeping genes change with disease and are therefore unsuitable for the normalization of such data. Melt curves were obtained after each qRT‐PCR to confirm single PCR products. Details of the qRT‐PCR primers used are provided in Table S2. All samples were examined in triplicate (Figures 8 and 9).

### 2.16 Statistical methods

All statistical analyses were performed using Stata 14. Histopathological scores were analyzed separately and as a combined total score. All analyses were conducted in triplicate for each of the six tissue samples analyzed and expressed in data plots as mean values ± SDs. Mixed ordinal logistic models grouped by sheep with time and MSC injections as variables were performed and, if significant, differences between treatment groups and times tested were assessed using Mann‐Whitney U ranked tests. The Benjamin‐Hochberg false‐positive correction for multiple tests was performed and gave a corrected *P* value of 0.045 for significance at an alpha value of 5%. Data were presented either as histograms of Mean ± SD or as box plots with median, 25 and 75% percentiles and range shown.

## 3 RESULTS

The procedures undertaken are summarized in Figure 1.

### 3.1 Demonstration of MSC multipotency

**Figure 2:**
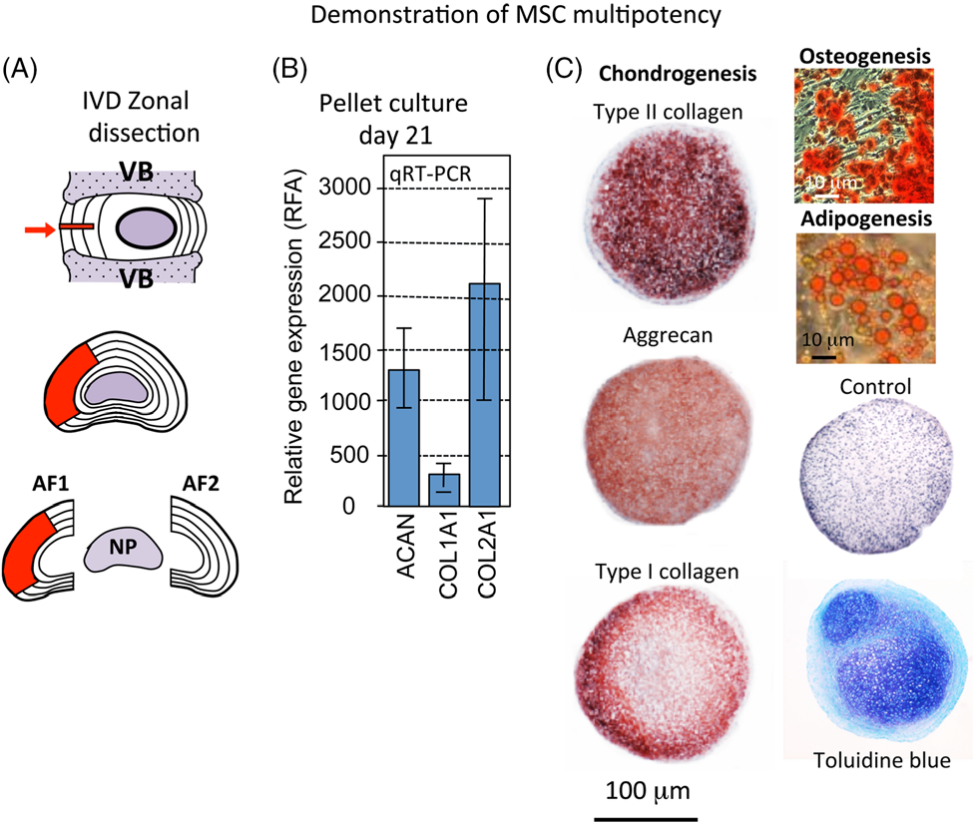
(A) Diagrammatic depiction of the location and size of the antero‐lateral annular lesion used to induce disc degeneration and Zonal dissection scheme demonstrating the AF zones 1 and 2 and NP used for the analyses undertaken in this study. (B) RT‐PCR data demonstrating chondrogenisis of MSCs in micromass pellet culture. (C) Demonstration of the pluripotency of the MSC preparation used in this study

Chondrogenic, osteogenic and adipogenic differentiation of the bone marrow derived stem cell preparation was demonstrated (Figure 2B,C) using appropriate selection medias. Bone marrow derived stem cells were grown in micro‐mass pellet cultures for 21 days. Isolation of total RNA and qRT‐PCR demonstrated *COL2A1* and *ACAN* gene expression, and the presence of toluidine blue stained GAG in pellet sections (Figure 2B). Deposition of calcium in monolayer MSC cultures identified by Alizaran red staining and oil droplets by Oil red‐O staining demonstrated osteogenic and adipogenic differentiation, respectively by the MSCs (Figure 2C).

### 3.2 Biochemical compositional analysis of IVD tissues

**Figure 3:**
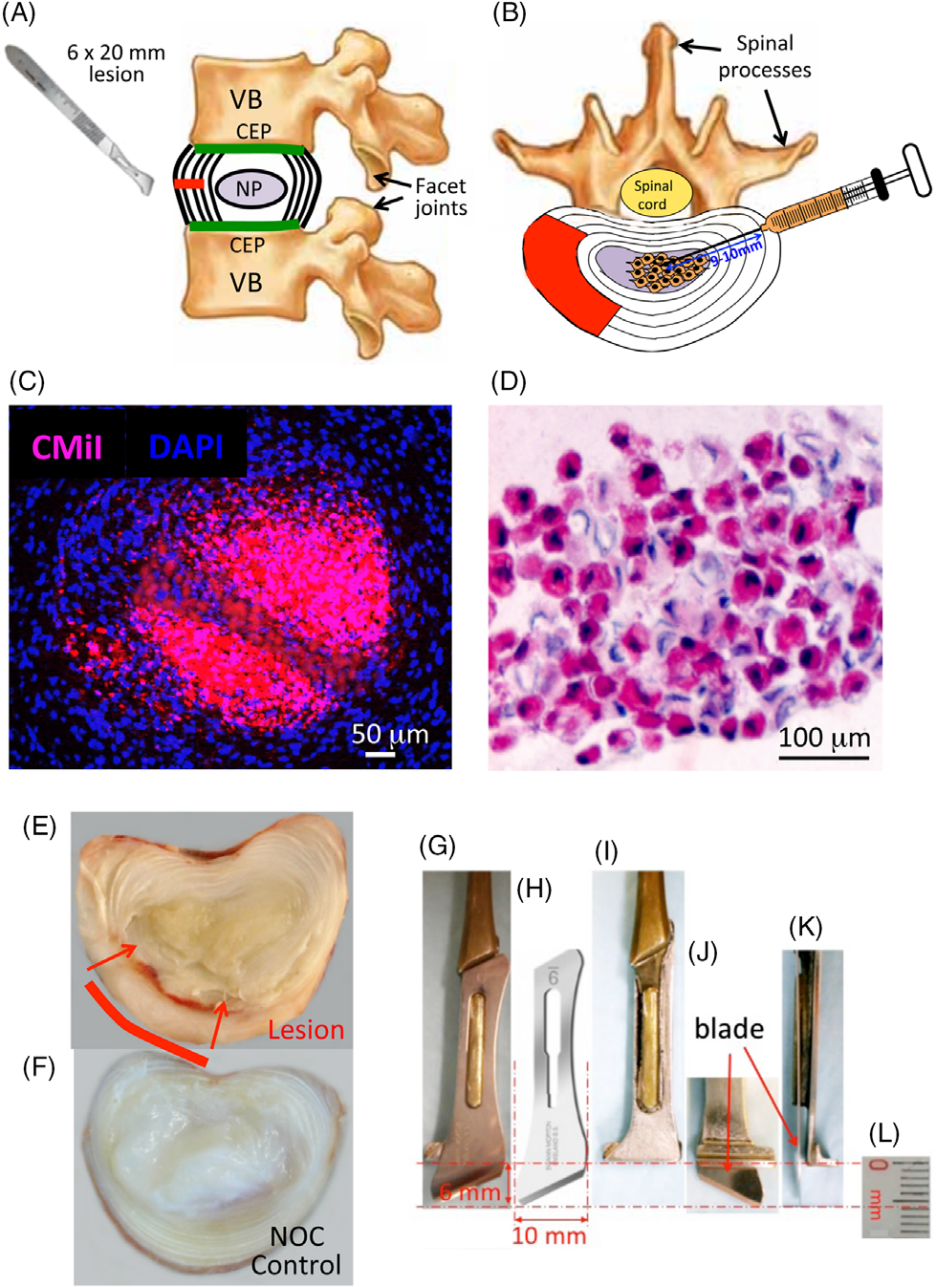
Diagram of the annular lesion site and adjacent spinal structures, vertebral body (VB), cartilaginous endplate (CEP), facet joints and spinal processes, spinal cord in the spinal canal. (A) The spinal injection site in the contralateral AF away from the annular lesion site for the intradiscal administration of MSCs into the NP (B). Demonstration of the localization of CMiI fluorescently labeled MSCs delivered into the NP following intadiscal injection (C), and in a nonstained section of NP in a bright‐field view (D). The stem cells are stained red by CMiI and thus can also be viewed by bright‐field microscopy. Macroscopic view of a horizontally bisected lesion IVD 1 month after establishment of the lesion. (E). The inner margins of the lesion are evident under naked eye observation by the penetration of blood vessels into the AF visible macroscopically. A nonoperated (NOC) disc is also shown for comparison (F). Details of the customized scalpel handle (G, I, J, K) and Abbott and Mann no 9 scalpel blade used to make the 6 × 20 mm annular lesion (H). This blade which is 10 mm wide was used to make two edge by edge incisions 10 mm wide incisions. The stop on the scalpel handle (G, I) allowed a maximum penetration depth of 6 mm as depicted in segments J, K showing a blade attached extending 6 mm past the stop on the scalpel (J, K). A scale bar (mm units) is also shown

**Figure 4:**
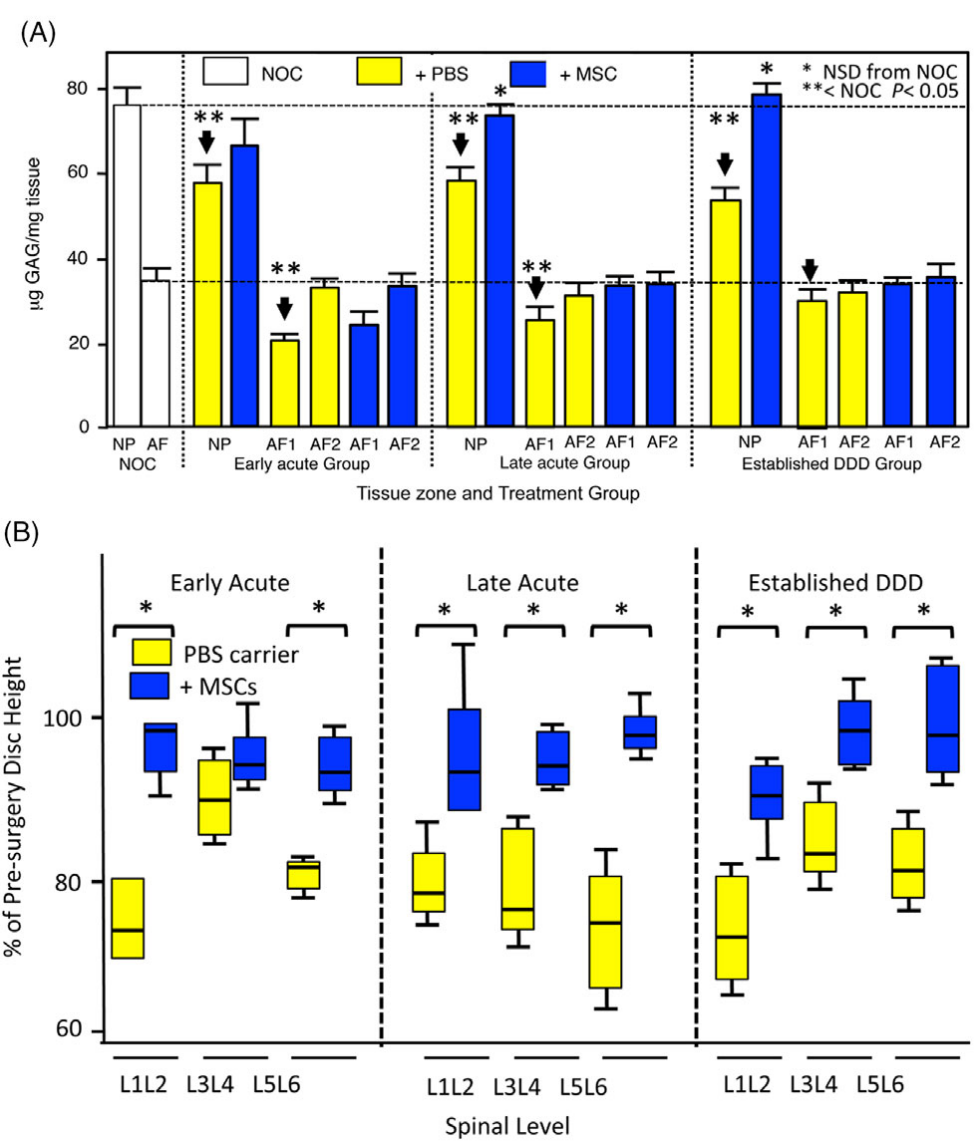
Histogram demonstrating the zonal GAG analyses of AF zones 1 and 2 and NP from the three treatment groups. (A). Box plots depicting disc height measurements of PBS carrier and MSC injected IVDs from the early Acute, late Acute and established treatment groups. Median values are depicted by a horizontal line within the boxes, 25% and 75% percentiles are also shown and ranges by the whiskers (B). Asterisks signify that MSC data was statistically different from the corresponding PBS injected data sets (*P* < 0.05). The analyses are based on six tissue samples in each case

Analysis of lesion zone GAG levels showed a significant reduction in the AF1 lesion zones of the EA and LA groups but not in the EST treatment group (Figure 3). A significant reduction in NP GAG levels in the lesion affected IVDs was normalized by MSC injection in all three treatment groups (Figure 4A). Tissue hydroxyproline contents in AF and NP were largely unchanged compared to the NOC levels and were unaffected by the MSC treatment, with the exception of the LA group where the hydroxyproline content was decreased in the MSC discs in both AF zones (data not shown).

### 3.3 Radiographical changes in lesion IVDs

Disc heights were measured from longitudinal lateral plain radiographs of the lumbar spine prior to surgery and at the end of the study (Figure 4B). Loss of disc height was evident in lesion discs treated by PBS injection, with a 15% to 25% reduction compared to pre‐surgery values at every disc level. MSC‐injected IVDs re‐attained 92% to 95% of pre‐lesion disc heights.

### 3.4 Histological examination of IVD tissue sections

**Figure 5:**
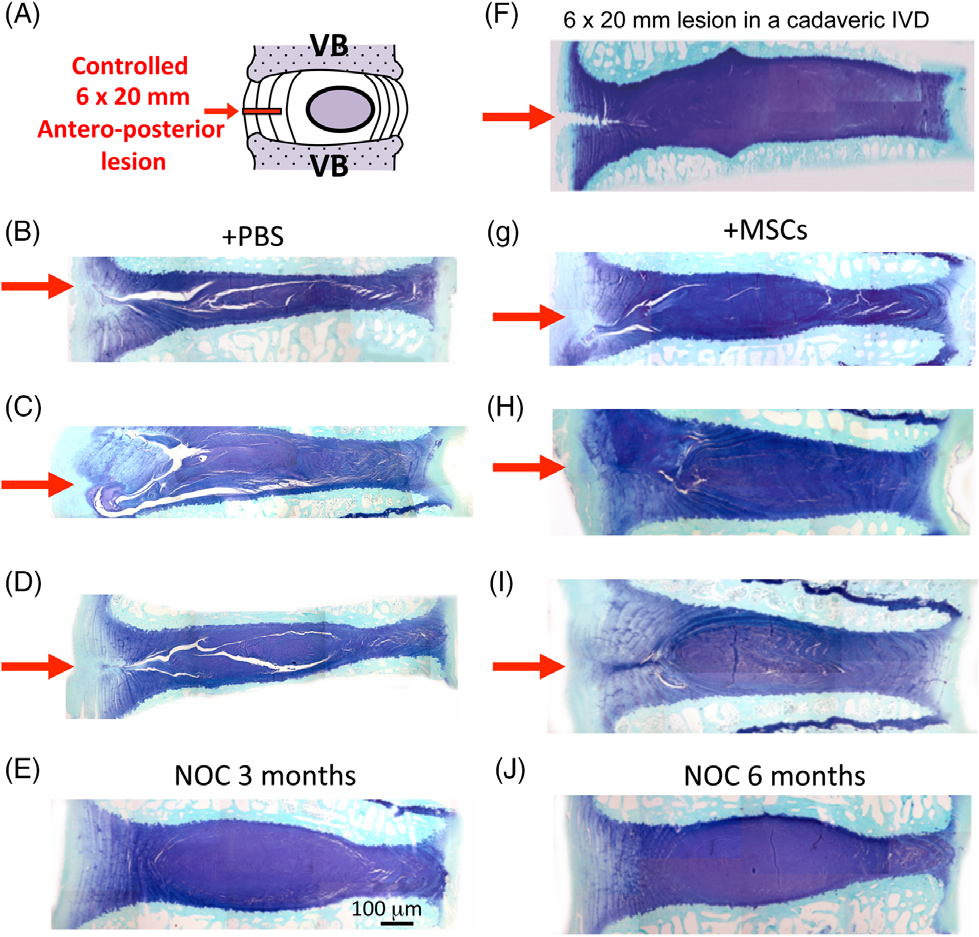
Diagrammatic depiction of the lesion site and adjacent discal structures (A). Toluidine blue‐fast green stained vertical sections of IVDs and adjacent vertebral bodies of lesion affected IVDs (B, C, D; G, H, I) and nonoperated control (NOC) IVDs (E, J). PBS carrier injected IVDs from the early Acute (EA) (B), late Acute ([LA)] (C) and established treatment groups [EST](D) and corresponding MSC injected IVDs from the EA (G), LA (H) and EST groups (I). Notice the reduced disc height and prominent lesions of the PBS carrier injected IVDs on the left hand side and near normal disc heights and significantly reduced lesions in the MSC treated IVDs on the right hand side of the figure. The lesion site is shown with a red arrow. A freshly made lesion in a cadaveric disc is shown depicting the initial extent of the lesion (F). See also Figure SS7 for further examples of MSC treated IVDs from each treatment group. Lesion induction was performed for 4 weeks in the EA and LA groups and 12 weeks in the EST group. The images shown were prepared from tissues which had undergone an 8 or 22 week recovery period (EA and LA groups) or 14 weeks (EST group)

Histological inspection of toluidine blue stained IVD sections showed that GAG levels were reduced in lesion IVDs injected with PBS carrier only but were significantly higher in the MSC treated IVDs (Figure 5 compare plates B, C, D with G, H, I) and IVD heights were also restored. The original IVD lesion propagated through the IVD towards the contralateral AF in the PBS injected IVDs but was largely repaired in the MSC treated IVDs (Figure 5G‐I).

### 3.5 Biomechanical assessment of lesion IVDs and the effect of MSC injection

**Figure 6:**
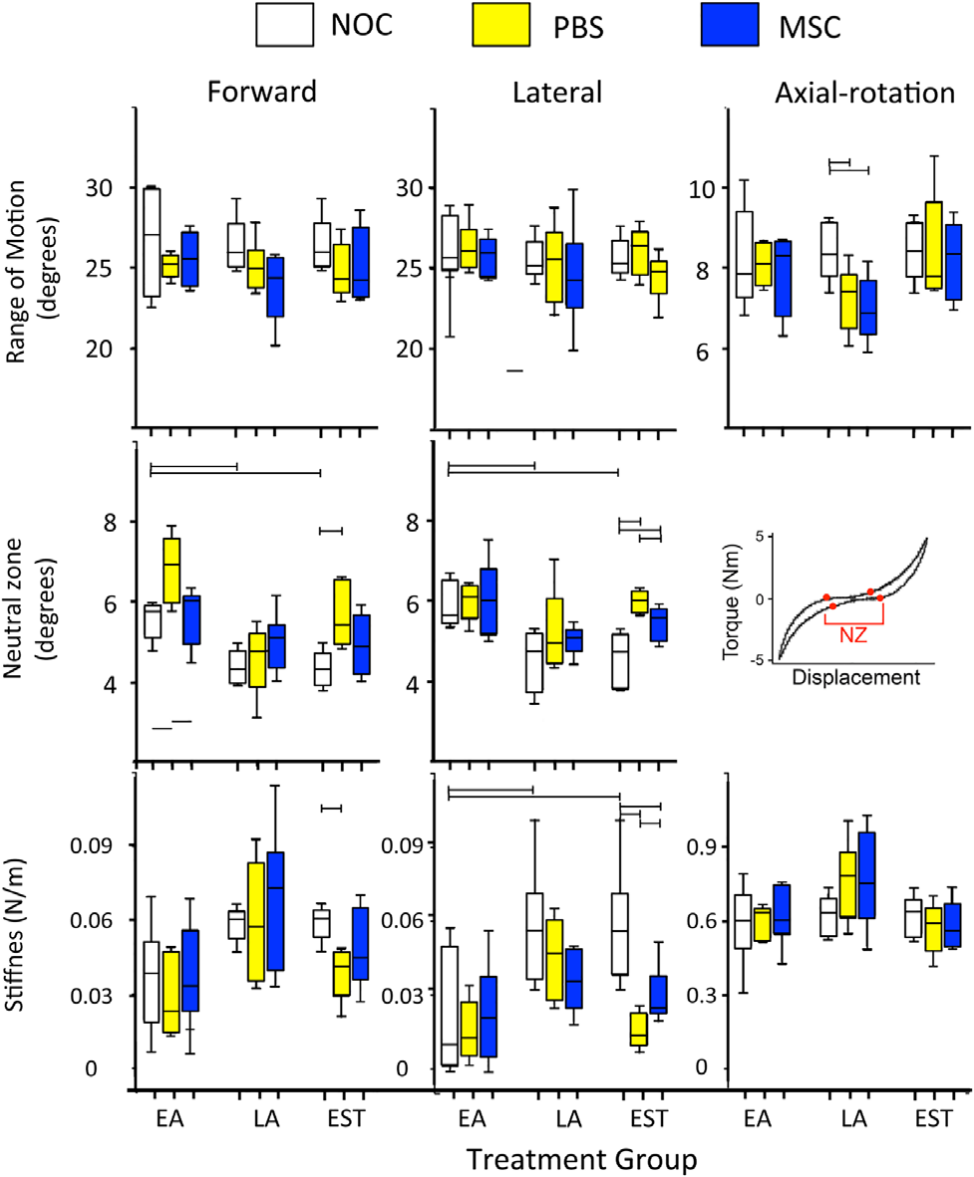
Biomechanical testing of IVDs from the early Acute early (EA), late Acute (LA) and established (EST) treatment groups. Horozontal bars represent statistical comparisons between data sets that were made. Six tissue samples were tested in each case and mean values ± SDs plotted. Lesion induction was performed for 4 weeks in the EA and LA groups and 12 weeks in the EST group and which had undergone an 8 or 22 week recovery period (EA and LA groups) or 14 weeks (EST group)

The range of motion (ROM), neutral zone (NZ) and stiffness of the lesion discs in flexion/extension, lateral bending and axial rotation are shown in Figure 6. ROM was not different between treatments (NOC, PBS or MSC) or groups (EA, LA, EST), except in LA where there was a significant loss of ROM after surgery in both PBS and MSC discs in axial rotation. In the NOC groups, there was a significant age‐associated reduction in NZ (short vs long term) in both flexion/extension and lateral bending. There was no NZ in any specimens in axial rotation because there was no region of low stiffness. In both flexion/extension and lateral bending no difference was observed between treatments in the LA group. In the EA group in flexion/extension, the NZ of PBS‐discs increased from NOC levels but did not reach statistical significance (*P* = 0.056). In the EST Group, lesion disc NZ was significantly increased compared to NOC. When treated with MSC, the EA NZ appeared normalized in flexion/extension but was not significantly reduced compared to the PBS injected IVDs (*P* = 0.056). In the lateral direction neither the EA or LA groups demonstrated any change in NZ compared to NOC. MSC injection in the EST group significantly reduced NZ compared to PBS injected IVDs but remained greater than in NOC IVDs.

Disc stiffness increased with age in both flexion/extension and lateral bending (*P <* 0.05 in lateral direction only) but not in axial rotation. IVD stiffness did not alter with disc lesions or MSC injection in both Acute groups. Changes were seen in the EST group with a significant drop in stiffness in the PBS‐injected lesion discs compared to NOC IVDs in both flexion/extension and lateral bending. MSC treatment caused an increase in stiffness in the lateral direction compared to PBS injected IVDs (*P* < 0.05) and this was reduced compared to NOC IVDs (*P* < 0.05).

### 3.6 Histopathological scoring of IVDs

**Figure 7:**
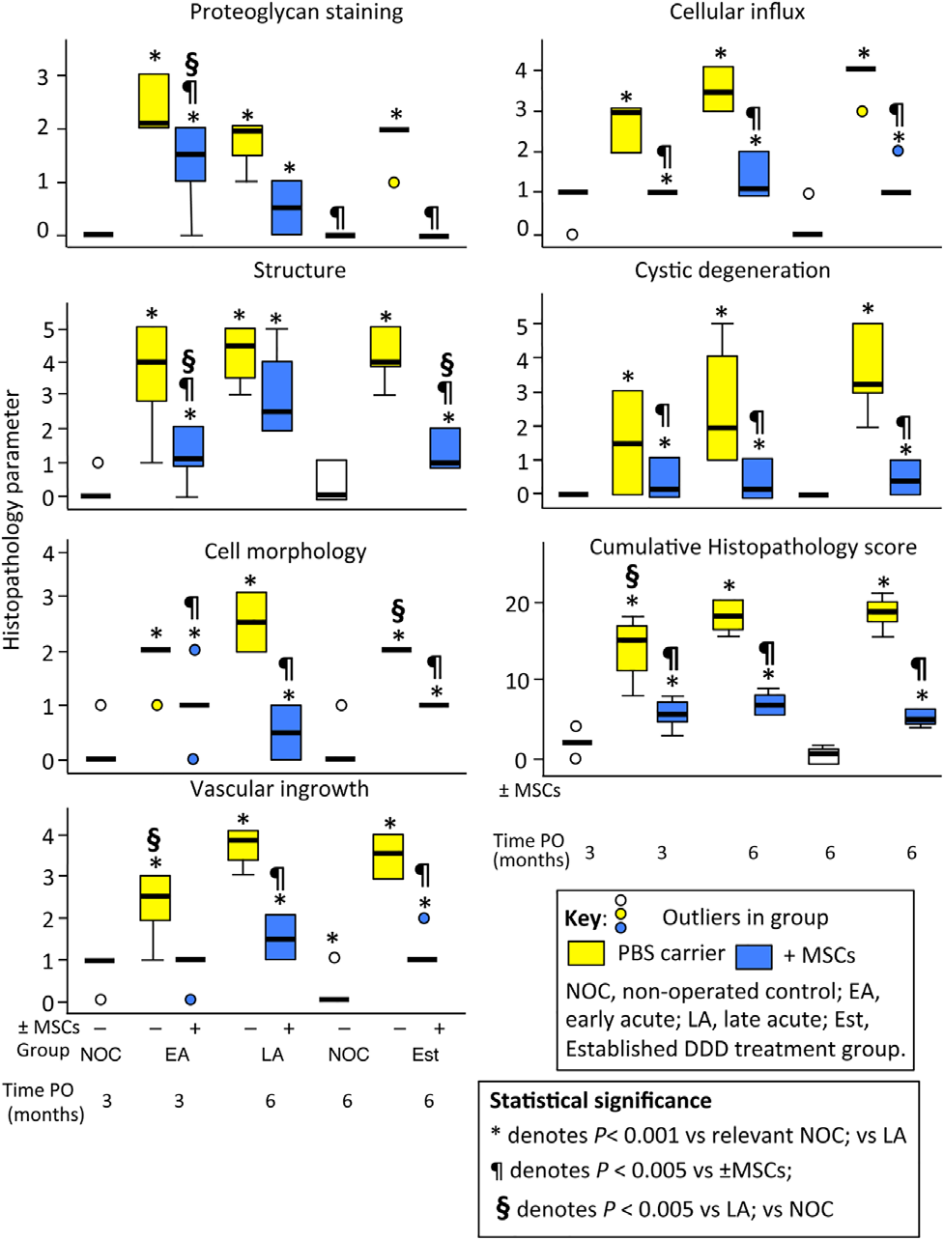
Histopathological scoring of IVDs from the nonoperated control (NOC), early Acute (EA), late Acute (LA) and established (EST) treatment groups. The box plots depicted represent 25/75% percentiles, median values are indicated by horizontal lines, ranges are represented by the whiskers. For explanations and histological examples of the descriminative criteria scored see Table SS1, and Figures SS2–SS8. Lesion induction was performed for 4 weeks in the EA and LA groups and 12 weeks in the EST group. The tissues which were scored had undergone an 8 or 22 week recovery period (EA and LA groups) or 14 weeks (EST group)

The advanced lesion development of the PBS carrier injected IVDs was reflected in higher histopathology scores (Figure 7). Cystic degeneration and chondroid metaplasia were more advanced in the PBS injected lesion IVDs. The significant reduction in cumulative histopathology score for MSC treated IVDs from 22 ± 2.2 to 4 ± 0.4 at study completion compared to PBS treated IVDs where cumulative score remained at 19 ± 2.5 was convincing evidence of the efficacy of MSC treatment.

### 3.7 qRT‐PCR gene profiling of IVD tissues

**Figure 8:**
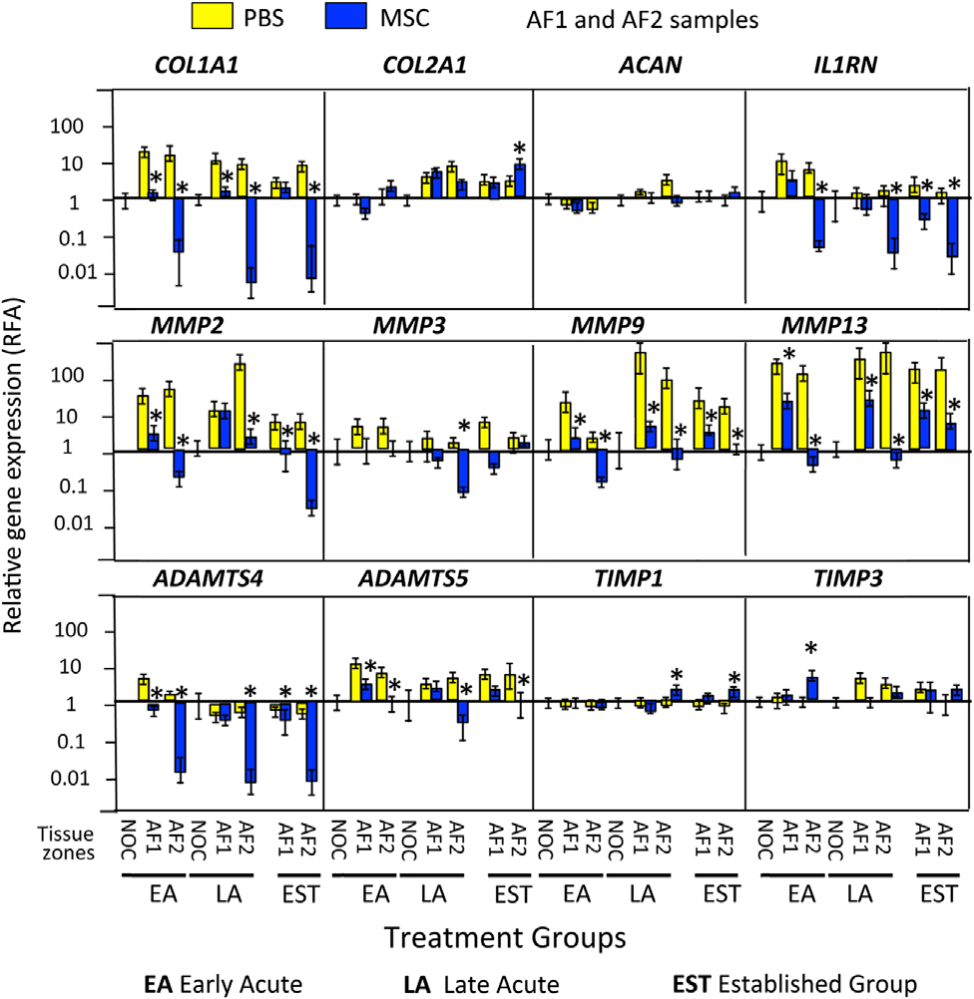
qRT‐PCR profiles of selected ovine IVD genes in the early Acute (EA), late Acute (LA) and established (EST) treatment groups in the AF1 (lesion), contralateral AF2 zones, and nonoperated control (NOC) IVDs. . Asterisks signify that MSC data was statistically different from the corresponding PBS injected data sets (*P* < 0.05). Six tissue specimens were analyzed in triplicate for each sample and means calculated. A grand mean of all six tissues ± standard deviations was plotted. Lesion induction was performed for 4 weeks in the EA and LA groups and 12 weeks in the EST group. The data shown is from tissues which had undergone an 8 or 22 week recovery period (EA and LA groups) or 14 weeks (EST group)

**Figure 9:**
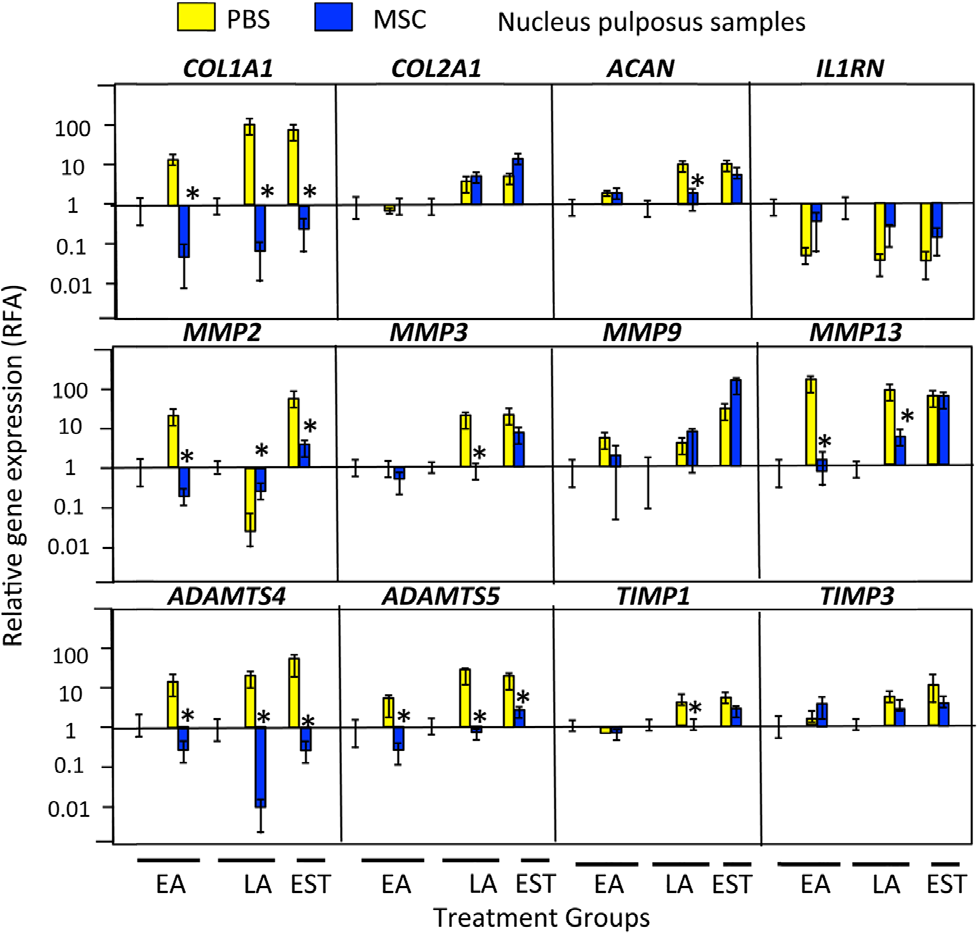
qRT‐PCR profiles of selected ovine IVD genes in the early Acute (EA), late Acute (LA) and established (EST) treatment groups in the NP zones, and nonoperated control (NOC) IVDs. Asterisks signify that MSC data was statistically different from the corresponding PBS injected data sets (*P* < 0.05).). Six tissue specimens were analyzed in triplicate for each sample and means calculated. A grand mean for all six tissues ± standard deviations was plotted. Lesion induction was performed for 4 weeks in the EA and LA groups and 12 weeks in the EST group. The data shown is from tissues which had undergone an 8 or 22 week recovery period (EA and LA groups) or 14 weeks (EST group)

*COL1A1* levels were significantly down‐regulated and *COL2A1* and ACAN maintained in the MSC treated IVDs. *IL 1RN, MMP2, 3, 9, 13* and *ADAMTS4, 5* were all down‐regulated while *TIMP1* and *TIMP3* levels were essentially unaffected in the MSC treated IVDs, contrasting with the PBS carrier treated lesion IVDs where most of these genes were up‐regulated (Figures 8 and 9). Collectively this gene expression data explains why the annular lesion propagated in the PBS carrier injected IVDs and did not undergo repair, and when the up‐regulation in anabolic matrix genes is also taken into account explains the annular repair evident in the MSC treated IVDs.

**Figure 10:**
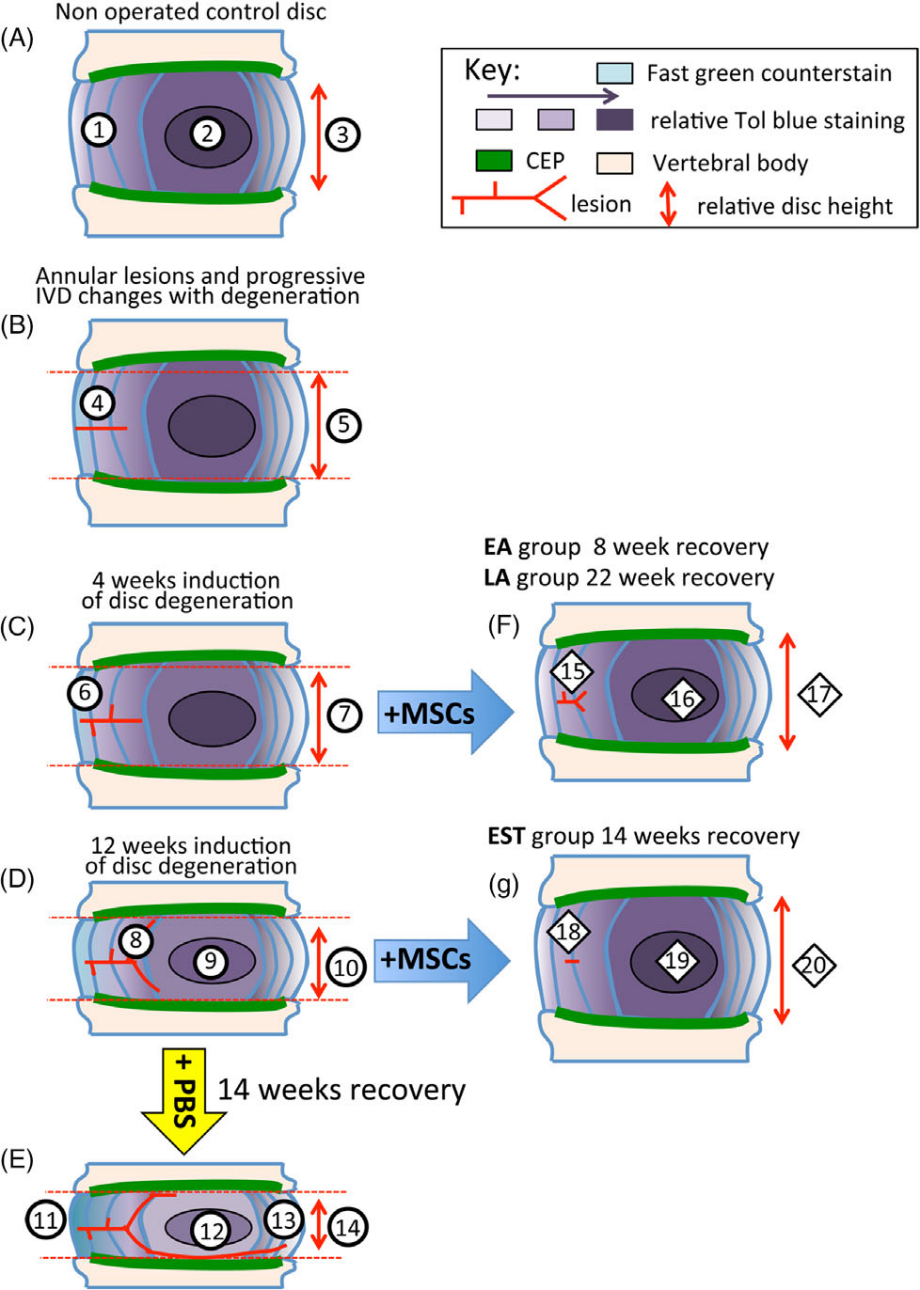
Diagrammatic depiction of sheep IVDs undergoing degeneration and recovery following MSC administration. (A) Control NOC disc, (B) establishment of lesion, (C) changes to IVD and lesion site 4 weeks after induction of disc degeneration. (D) Changes to IVD and lesion site 12 weeks after induction of disc degeneration. (E) Further progression of disc degeneration and lesion development after injection of PBS carrier after 14 weeks recovery. (F) Reversal of degenerative features by MSCs in IVDs of EA and LA treatment groups. (G) Reversal of degenerative features by MSCs in IVDs in EST treatment group. EA, early Acute; LA, Late Acute and EST, established treatment group; NOC, nonoperated control. Explanation of labeled features. *Typical features of a normal nonoperated control (NOC) IVD.* (1) Normal AF containing a gradient of toluidine blue staining. (2) Localization of toluidine blue staining in the nucleus pulposus (NP). (3) Normal disc height. *Progressive features evident as IVDs undergo degeneration induced by a controlled annular defect.* (4) Establishment of the controlled outer annular surgical defect. (5) Slight reduction in disc height. (6) After 4 weeks induction of disc degeneration de‐lammellations are generated by the defect. (7) Further reduction in disc height with advancing disc degeneration and decreased toluidine blue staining in the NP. (8) Bifurcation of the defect in the inner AF. Focal proteoglycan loss along the tract of the lesion in the outer AF. (9) Further reduction in toluidine blue staining in the NP. (10) Further reduction in the disc height at 12 weeks induction of disc degeneration. *Features in IVDs which received injection of PBS carrier rather than MSCs resulting in degenerative changes in the IVD over the next 14 weeks.* (11) Thickening of outer AF lamellae devoid of proteoglycan staining but loss of normal lamellar organization. (12) Reduction in the proteoglycan content of the NP. (13) Propagation of the outer annular defect towards the contralateral AF. (14) Significant reduction in disc height. *Reparative changes in IVDs induced by intradiscal administration of MSCs into IVDs that had undergone disc degeneration for 4 weeks after 8 or 22 weeks recovery with MSCs.* (15) Significant reduction in lesion size with a residual lesion still evident. Repair of the outer AF. (16) Proteoglycan content of the NP largely replenished. (17) Recovery of close to normal IVD heights similar to NOC IVDs. *Reparative changes in IVDs induced by intradiscal administration of MSCs into IVDs that had undergone degeneration for 12 weeks and had a 14 week recuperative period.* (18) Recovery of AF proteoglycan levels, almost complete disappearance of annular lesion. (19) Recovery of normal proteoglycan levels in NP. (20) Re‐attainment of normal disc height

A diagrammatic representation of the lesion and MSC treatment groups and the main outcomes of this study is provided (Figure 10). A point by point summary of the major findings is also provided.

Supplementary figures are also provided to illustrate specific features of the structure and cellular morphology of the normal and degenerate intervertebral discs and to demonstrate features which were used in the histopathological scoring of disc tissues. Table S1 provides the discriminative criteria assessed during histopathological scoring. These terms are described further elsewhere.^52^

Figure S2 describes the normal cellular morphology of the AF and NP and cell cloning/clustering observed in the AF in degenerate IVDs.

Figure S3 provides examples of the chondroid metaplasia (A‐E) and cystic degeneration (F, G) evident in lesion affected IVDs. Chondroid tissue forms as part of an attempted annular repair process. In some cases the chondroid tissue mass is integrated to a large degree with the annular lamellae (B, C) while in others the chondroid tissue is evident as an island of cartilage like tissue isolated from the surrounding inner annular tissue (D, E). The cells within the chondroid tissue have typical chondrocytic morphologies and are surrounded in a basophilic cartilage like extracellular matrix (A). Cystic degeneration was also observed in occasional degenerate IVDs (F, G). Cysts deficient in proteoglycan were typically located adjacent to the CEPs.

Figure S4 illustrates the cell clustering frequently observed in the inner and outer AF (A, B) associated with lesion propagation through the annulus. Ingrowth of blood vessels was also prominent features along the track of the annular lesion in degenerate IVDs (D, E). Figure S5 shows the cell morphology of the normal AF (A, B) and NP (A), occasional doublet cells were observed in the NP (arrows in A). Figure 6, illustrates features of the annular lesion as it propagates through the inner AF, around the NP towards the contralateral AF. The outer AF lesion track displays a focal depletion of proteoglycan evident as intensely fast green stained areas in the outer AF (1), bifurcation of the lesion (2) and de‐lamellation (3) were also frequently observed in lesion affected IVDs. Advanced development of the lesion was also associated with a significantly reduced disc height (E). Figure S7 provides additional examples of annular repair and regeneration of the IVD in each of the MSC injected treatment groups. Injection of PBS rather than MSCs resulted in the propagation of the annular lesion, depletion of proteoglycan and a severe reduction in disc height. Chondroid cell nests have also been observed in the normal ovine IVD (Figure S8A‐C). These were present in a dense basophilic proteoglycan rich matrix dissimilar from the matrix surrounding cell clusters in the vicinity of annular lesions (Figures [Link], [Link]). Resident cells in the NP also had a dissimilar morphology to these groups of cells within these cell nests (Figure S8 D, E).

## 4 DISCUSSION

### 4.1 Optimization of the annular lesion for the induction of disc degeneration

In an earlier pilot study, we evaluated a series of progressively deeper and wider annular lesions in our ovine model with a view to producing a destabilizing lesion which would produce accelerated disc degeneration but without prolapse of the NP through the residual AF of the lesion site. A 6 mm deep 20 mm wide defect was subsequently selected and shown to produce disc degeneration in a 3 to 6 month experimental period.^53^ To simplify production of a reproducible annular defect customized scalpel handles and scalpel blades were used as outlined in the present study.

### 4.2 Selection of the most appropriate MSC injection number to effect a therapeutic response in degenerate IVDs

When deciding on the most appropriate number of MCs for intradiscal injection we evaluated cell numbers which had been used in earlier studies (Table 1). We subsequently selected an injection number of 10 million cells per disc. This was similar to cell numbers employed in a number of human clinical trials for the treatment of disc degeneration and the alleviation of low back pain (Table 2).

### 4.3 Patient selection for prospective therapeutic treatment

An important consideration in any prospective treatment is whether it will be effective during early acute and later acute stages of the disease process as well as in established chronic stages of degeneration. In order to address some of these questions we utilized three treatment groups in our ovine model, to simulate early and late acute and established stages of the disease process administering MSCs at early and later stages of IVD degeneration. All three MSC treatment groups displayed beneficial effects in terms of disc repair and a significant reduction in cumulative degenerative histopathology score (*P* < 0.001). The EST treatment group had a marginally greater reduction in histopathology score than the other treatment groups, possibly reflecting the more advanced disease which develops in this group with the longer lesion induction period however there was not much clinical difference between the treatment groups. There was no added benefit in a longer recuperative period of 22 weeks in the LA treatment group over a shorter one of 8 weeks for the EA or 14 weeks for the EST group. Thus early beneficial effects using MSCs were sustained to later time points. A PET imaging study^19^ has shown MSCs remain viable in canine IVDs for 3 weeks thus all treatment groups were expected to display beneficial responses from the MSC injections used.

### 4.4 The multi‐disciplinary design of the present study

A major strength of the present study lies in its multidisciplinary experimental design, use of a well established validated large animal model of disc degeneration which closely reproduces the pathobiology of disc degeneration in humans^53^, ^60^ and the use of an experimental design which allows direct comparison of the efficacy of MSC treatment at different stages of IVDD. Spinal manipulation studies in sheep further demonstrate that annular lesions perturb normal spinal biomechanics, neurophysiology, stabilization of vertebral lumbar motion segments and muscular contributions to dynamic dorsoventral lumbar spinal stiffness^61^, ^62^, ^63^, ^64^

No other model of disc degeneration has received such a comprehensive appraisal as the ovine model. The DDD which develops in the sheep model also effects multifidus muscle remodeling that may also contribute to LBP.^65^, ^66^ Experimental DDD changes multifidus pro‐inflammatory cytokine gene expression profiles^67^, ^68^ and mimics LBP changes in human spinal tissues.^69^ Macrophages and TNF have active roles in the subacute/early chronic phase of remodeling in muscle, adipose and connective tissues of the multifidus muscle during IVD degeneration and represent a novel therapeutic target. Although MSC treatment prevents fatty infiltration and fibrosis of the multifidus muscle after the development of an IVD lesion, it cannot prevent a muscle inflammatory response and muscle fiber transformation. These findings highlight the potential role of MSC therapy after IVD injury, but indicate other interventions may also be necessary to optimize recovery of spinal muscles such as the multifidis.^70^, ^71^

Traumatic loading generates discal lesions through cumulative stress fractures of fibrillar components which attach the human IVD to the ring apophysis of the vertebral body. Similar changes have also been observed in the ovine model.^72^ Separation of Sharpeys fibers generates rim‐lesions pre‐disposing the NP to degeneration^73^ and these propagate through the AF resulting in radial and circumferential fissures, separation of annular lamellae (de‐lamellation) and degenerative changes in the NP and CEPs.^74^ These changes include a loss of IVD proteoglycan and tissue hydration,^75^ a reduction in disc height, changes in endplate vascularity^76^ and vertebral bone density adjacent to the annular lesion^77^ and osteoarthritis of the facet joints.^78^ An increase in blood vessel and nerve in‐growth^79^ and an influx of cells expressing FGF‐2 and TGF‐β1 in the lesion affected IVD^80^ also occur in the ovine model compromising normal IVD biomechanical properties and are hallmarks of IVD degeneration in man.^12^ The aggressive 6 mm deep and 20 mm wide annular lesion used in the present study accelerates disc degeneration over that seen previously with the Osti model^60^ which can take up to 18 months for degenerative changes to develop. The re‐attainment in functional properties observed in the present study is therefore a particularly significant finding. No other large animal model has used such a large defect to induce IVDD. The recovery of disc composition, disc height and normalization in biomechanical properties observed are noteworthy findings and strong evidence of the efficacy of MSCs for discal repair. Earlier findings with small animal models of DDD using small needle puncture defects cannot be considered to provide as convincing evidence of IVD repair and restoration of function.^81^ The focal loss of GAG from the lesion site and associated NP degeneration observed in the present study was countered by administration of MSCs with GAG levels returning to levels found in age matched NOC IVDs by the end of the treatment period. A reduction in disc height with lesion development and normalization by MSC treatment was consistent with initial reduction in IVD material properties and a return to normal IVD material properties. Histopathological scoring of lesion and MSC treated IVDs supported this positive gain in tissue function. qRT‐PCR of *COL1A1, COL2A1*, *ACAN*, *MMPs*, *ADAMTS4, 5* and *TIMP1, 3* explained the enhanced ECM production and decreased IVDD and positive repair response. A prominent feature of MSC treatment was the lack of a fibrotic response consistent with the reduced fibrosis and hypertrophic scar formation reported in a number of studies^82^, ^83^, ^84^, ^85^ including the IVD.^86^ This is considered to occur through the regulation of immunomodulatory processes^50^, ^87^, ^88^ by the MSCs suppressing inflammation^50^ and TGF‐β mediated cellular responses^84^, ^85^ which otherwise can lead to excessive laying down of collagen as part of a repair response. We have observed previously in the ovine annular lesion model of disc degeneration that an influx of cells to the annular lesion site occurs, these cells expressed TGF‐β to a fibrotic response which was observed in the lesion site.^80^

### 4.5 Mode of action of MSCs

Secretion of paracrine factors is now recognized as the primary mechanism by which MSCs induce a regenerative environment to promote tissue healing,^89^ cell‐to‐cell contact has been shown to be of some importance but may not be an essential component.^90^, ^91^ MSCs home to sites of inflammation where they secrete soluble trophic factors such as growth factors, cytokines, and chemokines to promote tissue repair.^92^ In‐vivo studies show MSC therapy promotes angiogenesis and growth and differentiation of local progenitor cells, prevents fibrosis and apoptosis, attracts immune cells to injury sites, and are immunomodulatory in the local environment.^92^, ^93^, ^94^, ^95^ Engraftment of MSCs appears to be unnecessary for a therapeutic effect, and MSCs likely persist through the initial inflammatory phase into the repair and remodeling phases of tissue healing. Adult MSCs from bone marrow, peripheral blood, or adipose tissue, have been or are currently being investigated in over 600 clinical trials in musculoskeletal diseases, degenerative and traumatic neurological diseases, and immune mediated diseases.^96^ MSC therapy has been effective at treating several animal models of disease^97^, ^98^ and shown success in human clinical trials.^96^ Supplemental figures are supplied to document the range of degenerative features we observed in the present study and to demonstrate the efficacy of MSCs for discal recovery (Figures [Link], [Link]).

### 4.6 Chondroid metaplasia and cystic degeneration in the ovine large lesion DDD model

Extradural cysts similar to the intradiscal cysts observed in the present study have also been observed in the elite athlete.^99^ These typically result in compression of spinal ganglia and neurological deficits. IVD cystic degeneration can be imaged by MRI.^100^ Cystic degeneration was only observed in the PBS injected IVDs in this study.

Chondroid metaplasia was originally described in the classical work of Hansen (1952)^101^ in the nonchondrodystrophic (non‐ChD) canine^102^, ^103^ and the lapine IVD.^104^ Chondroid metaplasia is considered to be a forerunner to the IVD calcification frequently found in ChD canine breeds.^101^, ^105^ Calcification of the ovine IVD has also been observed^106^ suggesting that the ovine merino could be considered a mildly ChD breed. Like the beagle where an inbuilt form of dwarfism has produced characteristic changes in the long bone growth plates,^107^ the merino was also originally bred as a long‐legged form. A Rambouillet serving ram from France had a major influence on the original Macarthur sheep flock which formed the basis of the Australian merino breed characteristics.^108^ Over time this long legged trait has been bred out to a shorter legged “modern” fine wool merino. So‐called “Macarthur” sheep are named after General John Macarthur, an early Australian explorer and pioneer of the wool industry in Australia. The progeny of the original long‐legged breed form have been maintained and bred true as a Heritage flock in Australia held at Macarthur Agricultural College, Camden, NSW, Australia (http://www.heritagesheep.org.au). The chondroid tissue we observed along the track of the annular lesion was well integrated with annular tissues in some cases and may have contributed to the stabilization of this defect. However, cartilage is optimally designed to withstand compressive load and not the radial hoop stresses the annular lamellae encounter. These chondroid tissue masses may be a misdirected repair response by the administered MSCs but may over time undergo remodeling to a fibrocartilaginous tissue. Chondroid tissues were not observed in any of the PBS injected IVDs thus on this basis cannot be considered a degenerative feature.

### 4.7 Clinical trials for the use of MSCs in the treatment of disc degeneration and LBP

A number of preclinical and laboratory based studies and clinical trials have been or are currently being undertaken world‐wide using BMMSCs or ADMSCs to treat disc degeneration and alleviate LBP (Tables 1 and 2). Further clinical trials have been undertaken by ISTO Technologies in 2012 to 2016 in the USA using juvenile allogeneic articular chondrocytes (NCT01771471), and in 2013 by The Foundation for Spinal Research Education and Humanitarian Care Inc (USA) using autologous or allogeneic BMMSCs (NCT02529566). A clinical trial was conducted by Arhus University Hospital, Denmark using autologous BMMSCs (EudraCT 2012‐003160‐44), BioHeart (USA) (2014‐2017) used autologous ADMSCs (NCT02097862). Tetec AG (Germany/Austria) used autologous disc cells to treat disc degeneration. This study is due for completion in 2021 (NCT01640457, EudraCT 2010‐023830‐22).

### 4.8 The ovine large lesion model of disc degeneration and LBP

Of the animal models which have been used experimentally to produce disc degeneration^81^ the ovine large lesion model^53^ provides the most compelling reproduction of the pathobiological changes^52^ which occur when the human IVD degenerates. The efficacy of administered stromal stem cells in the reversal of degenerative discal pathology was also clearly established in the present study and has been quantitatively scored in a histopathological scoring scheme we have also developed.^52^ However an important aspect of such studies is also the assessment of pain generation and this is a particularly difficult parameter to evaluate in a meaningful manner in an animal model. In the absence of verbal communication, changes in animal behavior and alterations in posture or weight bearing of the experimental animal are possible means of assessing the generation of pain in such models. Even with the benefit of verbal commentary in the clinical assessment of human IVD degeneration, a specific nociceptive cause of LBP is rarely identified despite the impact of a biomechanically deficient IVD being clearly identifiable. The normal IVD is poorly innervated and ingrowth of nerves occurs with IVD degeneration.^79^ Paradiscal tissues such as the anterior and posterior longitudinal ligaments, facet joints and vertebral body are all richly innervated and all of these tissues are potential sources of nociception.^109^ LBP is an extremely common and debilitating condition experienced by people of all ages thus it is imperative that a better understanding of this disorder be achieved.^110^, ^111^, ^112^, ^113^ In 2015, the global point prevalence of activity‐limiting LBP of 7·3% indicated that 540 million people were affected globally by LBP at any one time, LBP is now recognized as the number one musckuloskeletal condition and cause of disability world‐wide.^11^, ^15^ The socio‐economic impact of LBP on quality of life, associated healthcare costs in its treatment and lost productivity are very significant issues.

As in any animal model, the lack of verbal communication with sheep in our case confounds the diagnosis and characterization of the experience of pain. However, since sheep possess the same neuronal pathways and neurotransmitter receptors as humans, it would seem reasonable to expect that their perceptions of painful stimuli would be similar to in humans. However even in humans, individuals display widely differing responses to how they tolerate and respond to pain and much of this is due to the mental strength of an individual leading to difficulties in the evaluation of pain responses even when verbal communication is possible. The innervation of the IVD in sheep and human IVD are similar and nerve ingrowth has been demonstrated in degenerate IVDs.^79^, ^114^, ^115^ Local anesthetics, opioids, and nonsteroidal anti‐inflammatory drugs used in humans are all effective in sheep. In the present study, evaluation of the flocking behavior, feeding habits and ambulation of the lesion and nonoperated control (NOC) sheep groups by experienced veterinary personnel did not demonstrate any obvious differences between these two sheep groups. Radio‐frequency identification (RFID) microchips with GPS monitoring and automatic data logging capability could be applied to accurately measure individual sheep activity profiles in open paddocks following disc degeneration in a longitudinal format and potentially could provide some important information. Software for whole body 3D motion analysis has been developed from animatronics and kinematics software used in the leisure film industry and could also be incorporated into force plate, gait analysis and biomechanics programs to fully assess sheep behavior. The Pressure Mapping, Force Measurement and Tactile Sensors which have been developed for the analysis of animal behavior and specific applications in clinical biorobotics may well need to be applied to systematically evaluate subtle changes in body language following spinal surgery in sheep. In other kinematics and gait analysis animal studies sophisticated methodology including the use of high‐speed photography were required to measure changes in body language so it is hardly surprising that we did not observe overt differences between NOC and operated sheep in our study by naked eye observation. The enthusiasm of lesion sheep for feeding in open pasture was not different from NOC sheep, no limb weakness was obvious in any sheep and no signs of distress were observed. Human facial recognition software and cloud based technology has also been applied to assess small animal behavior experimentally and is capable of automation.^116^, ^117^, ^118^, ^119^ This technology is also beginning to be applied in the identification of livestock in the rural sector and can provide invaluable information on animal appetite habits, water intake and has motion capture capability which can be used as a measure of the habits and health of stock on an individual animal basis. Such technology awaits application in laboratory based large animal studies but nevertheless represents an interesting development which may be useful in determining animal behavior and postural patterns of relevance to determining pain profiles. This could provide measures of any potential postural adaptations in response to painful stimuli and the impact of specific experimental procedures on behavioral responses in large animals such as the ovine model. The current inability to discern pain profiles in sheep in which disc degeneration has been induced must therefore be considered a limitation of this model. Animal models of pain reproduction through disc degeneration have been developed in mice and rats.^120^, ^121^, ^122^ The assessment of changes in gait and ambulation and their correlation with pain profiles are more easily monitored in these small animal species however the structural changes reproduced in IVD degeneration in rodents do not reproduce those seen in humans, furthermore there are other reasons why rodent discs are less suitable as a model of disc degeneration than the sheep (reviewed in^81^). Future improvements in the analysis of sheep kinematics as outlined above may well provide a means of determining correlative pain profiles in the large lesion sheep model of disc degeneration.

### 4.9 Annular repair studies and MSCs

Annular repair has been examined in a number of animal models. Zhou et al co‐cultured rat bone marrow and adipose derived Mesenchymal stem cells (BMMSCs and ADMSCs) with AF cells and showed that MSCs had directive roles over the annular cells.^123^ BMMSC and ADMSC AF cell co‐cultures were subjected to qRT‐PCR analysis. This showed an up‐regulation in selected AF marker gene expression. BMMSCs induced a statistically significant increase in type II collagen and aggrecan expression by 7 days of co‐culture indicating an early chondrogenic response and an increase in type I collagen expression on days 14 to 21 indicating a delayed fibrotic response. ADMSC co‐cultures however required a 21 day culture period before an elevation in type I collagen expression could be detected. Histological analysis and the use of the metachromatic GAG reactive dye 1,9‐dimethylmethylene blue demonstrated an elevation in the production of proteoglycans containing sulfated GAG in the co‐cultures. This study suggested that BMMSCs might be more appropriate for the promotion of cartilaginous repair of the NP while ADMSCs elicited a collagenous repair response that might be more suitable for repair of annular defects.

Xu et al created a 1 × 1 cm annular defect in goat IVDs and administered BMMSCs using a gelatin sponge impregnated with MSCs and platelet rich plasma (PRP) as a growth factor source into the defect site. The IVDs were examined 3, 6 and 12 weeks after creation of the defect. IVD tissue sections were stained with H&E to examine cellular morphology, Masson Trichrome for collagenous organization, Alcian blue‐periodic acid Schiff stain to identify proteoglycan deposition and type II collagen was immunolocalised. This study showed that BMMSCs and PRP increased cell proliferation in the defect site, increased collagen and proteoglycan deposition and promoted annular repair.^124^ Li et al induced AF degeneration in 6 month old rabbits by removal of 5 mL of the NP using a syringe, no actual defect in the AF was made. BMMSCs (50 μL of 2 × 10^5^ BMMSCs/ml) were administered into degenerate IVDs 2 weeks later. IVD heights were determined 2, 7 and 10 weeks after lesion induction and the affected IVDs were subjected to histopathological scoring. The authors considered BMMSCs were beneficial for what they considered to be annular repair.^125^ Pirvu et al used an interesting combination of BMMSCs seeded in polytrimethylene carbonate (PTMC) scaffold to produce annular repair tissue in a bovine organ culture annulotomy model under dynamic load for 14 days. The MSC‐PTMC composite was held in place using a polyester urethane membrane which was sutured over the annular defect. Furthermore, this stabilization of the annular defect was considered important for the prevention of nuclear prolapse through the annular defect. Implanted MSCs showed an upregulation in type V collagen, a potential AF marker, and anabolic matrix genes and downregulation in catabolic gene expression thus promoted annular repair processes.^126^

It is difficult to directly compare the positive findings of the present study using MSCs with these earlier studies since they did not undertake as extensive analytical studies on the lesion and repair tissues or their material properties. Additionally the sheep more closely models the human IVD in terms of structure and of the cell populations present and how they respond to an annular defect. Furthermore, these earlier animal models did not utilize as extensive an annular lesion as the present study and annular repair was evaluated over a relatively short recuperative period compared to the present study. This reflects badly on the situation in the human IVD and in our opinion the results of the present study are more compelling. Studies in rats and rabbits do not provide as strong evidence of the efficacy of MSCs for annular and discal repair as the sheep model although they are supportive of MSC efficacy.

Interesting recent developments in bioscaffold design have resulted in production of CS containing scaffolds with the ability to direct MSC cell differentiation in‐situ. in the context of tissue repair this can be used to promote the production of matrix components conducive to tissue repair.^127^, ^128^ These bio‐scaffolds have yet to be applied to disc and annular repair, however they have properties which would be useful in such applications and are worthy of further examination.

In an extensive review on the use of bioscaffolds and MSCs for annular repair Sherafi et al describe commercially available Inclose surgical meshes, Xclose suturing closures and Barricaid woven mesh barrier devices made from polyethylene terephthalate for the closure and containment of annular defects. Annular closure devices based on a temperature sensitive biodegradable shape‐memory polymer network^129^ and photo‐crosslinkable poly(trimethylene carbonate)‐based macromers for closure of ruptured IVDs have also been developed.^130^ Despite promising results with these annular containment products recurrent disc herniations still frequently occurred and were common causes of back pain. The poor healing potential of the AF negatively impacts on the repair of tears in IVDs stabilized using sutures or a surgical containment device, making the disc highly susceptible to re‐tearing. Vascular ingrowth and nociceptive nerves occur in annular defects but these do not penetrate into the NP which is an avascular and aneural tissue.^79^, ^114^, ^115^ Biological glues may also be useful in the sealing of natural and surgical annular wounds. A novel bio‐glue discovered in the Australian frog *Notaden benetti* is an adhesive exudate from the dorsal skin which ensures sexual union with the male for an extended period to ensure effective fertilization.^131^ Its molecular composition and mechanism of action^132^ has shown this protein based adhesive^133^ is nonimmunogenic, biocompatible, displays elastomeric properties similar to elastin and adhesive properties several‐fold that of fibrin glue. Frog glue has been used to re‐attach infraspinatus tendon in rotator cuff repair,^134^ and bucket handle meniscal tears.^135^, ^136^ New Zealand green lipped mussel and barnacle glues also have strong adhesive properties suitable for orthopedic applications, but await commercialisation.^137^, ^138^, ^139^, ^140^, ^141^

The low number of cells in the mature AF of advanced stages of senescence exclude them as a suitable cell source for the seeding of scaffolds. MSCs are a useful alternative cell type for annular repair and have been isolated from annular tissues of normal and degenerate IVDs.^142^, ^143^, ^144^, ^145^ The cell clustering seen in the vicinity of annular defects is evidence of resident stem cell activity in these tissues.^146^ The healing potential of the AF overall however is low with most of its intrinsic healing occurring at its outermost margins.^147^ Annular defects which penetrate into the mid and inner AF however do not undergo spontaneous repair and over time these propagate further into the disc and can lead to the development of rim‐lesions, de‐lamellations, bifurcations, and extension around the NP as far as the contralateral AF with the formation of radial and cirumferential tears.^74^ In the present study, the administered MSCs prevented the extension of the initial annular defect and also resulted in healing of the outer annular defect, a significant finding given the large lesion size used in this sheep model.

Reproducing the anisotropic annular structure is a prerequisite for an effective treatment.^148^, ^149^ No studies have so far demonstrated bioscaffolds with mechanical properties and structural features matching those of the native AF. Thus many questions and challenges still remain in the development of effective strategies that lead to regeneration of the damaged AF using a scaffolding approach. However, in the present study MSCs in isolation were effective in the repair of annular defects and biomechanical studies indicated that the MSC treated IVDs had re‐attained similar biomechanical characteristics as native IVD tissue. In contrast, in IVDs which received injections of PBS carrier, the controlled outer annular surgical lesion continued to propagate through to the inner AF and NP and the biomechanical properties of these tissues deviated markedly from those of NOC IVD tissues.

## 5 CONCLUDING REMARKS

As of May 2018, there were over 600 registered clinical trials examining the therapeutic potential of MSCs to treat a variety of pathological tissues with ~17 of these looking specifically at the treatment of intervertebral disc degeneration and low back pain. (https://clinicaltrials.gov/). Understanding the biology of MSCs is the first step to revealing their full therapeutic potential. In July 2018, the therapeutic goods administration (TGA) in Australia issued a change in the regulations governing the use of autologous human cell and tissue products for therapeutic procedures and the claims that the providers of such procedures can make regarding the efficacy of such interventions.^1^ The reason for this change in TGA guidelines was to address the growing global concerns with direct consumer advertising of unproven autologous stem cell interventions. Furthermore, in May 2018, the US Food and Drug Administration (FDA) filed injunctions in Federal Court with a number of stem cell clinics to cease marketing of unapproved unproven products. A Florida‐based stem cell clinic and a network of about 100 clinics in California are currently facing litigation brought by the FDA to cease the marketing of their products for the treatment of Parkinson's disease, amyotrophic lateral sclerosis, chronic obstructive pulmonary disease, heart disease and pulmonary fibrosis. This further reinforces the need to better understand the mode of action of therapeutic stem cells in tissue repair processes by undertaking basic preclinical and laboratory based studies such as the present study. *The clinical stem cell trials which have been conducted and are on‐going for the treatment of disc degeneration and the alleviation of low back pain, amply demonstrate the need for the perfection of such procedures however of equal importance is a fundamental understanding of how stem cells operate at the tissue repair level in such procedures. Our study provides strong evidence supporting the therapeutic use of stem cells for intervertebral disc repair and provides some insights into their mode of action. Further work however is still required to fully understand this extremely interesting and potentially very beneficial family of cell types.*

## CONFLICTS OF INTEREST

The authors have no conflicts or financial disclosures to make.

### Author contributions

C.C.S. was responsible for the day‐to‐day running of the study, biochemical, qRTPCR, stem cell culture laboratory experiments, data collection, contributed to manuscript writing and review. A.D., C.B.L. were responsible for animal surgeries, monitoring of animal recovery and welfare issues and both contributed to manuscript writing and had intellectual input into experimental design. R.B. assisted with animal surgery, undertook spinal imaging and data recording and manuscript review. E.C., J.M. and C.C.S. undertook the biomechanical testing of I.V.D. tissues and E.C. designed the test rigs. Supervised testing and interpretation of data and also contributed to manuscript writing. M.M.S. undertook the statistical analyses of all data and reviewed the manuscript. J.M. coordinated contributions from all authors, had intellectual input into experimental design and interpretation of data, wrote the manuscript and coordinated review changes and prepared the final submission. All authors approved the final version of the manuscript.

## Supporting information


**Figure S1.** Identification of CD34, CD45, CD90 and CD 105 expression by ovine bone marrow stromal stem cells and no staining for CD14, CD19, CD73 by flow cytometry.

**Figure S2.** Cellular morphology in the normal annulus and the dissimilar cell clusters observed in the mid and inner AF associated with annular lesion development in degenerate IVDs. H&E and toluidine blue‐fast green stained tissue sections.

**Figure S3.** Chondroid metaplasia (A‐E) and cystic degeneration (F, G) evident in lesion containing IVDs treated with MSCs. In some cases chondroid tissue along the tract of the annular lesion is integrated with the annular lamellae and appears to contribute to repair of the lesion (B, C) while in other samples this chondroid tissue occurs as an isolated tissue mass (D, E). Cells in the chondroid tissue have a typical rounded chondrocytic morphology surrounded by a proteoglycan rich cartilaginous ECM (A). Cyst formation in degenerate IVDs not treated with MSCs, typically occurring adjacent to the CEP (F, G). Toluidine blue‐fast green stained tissue sections. Tissue sections from EST MSC treatment group (A‐C), EA (D) and LA MSC treatment groups (E). Chondroid metaplasia only occurred in the MSC treated IVDs, this may represent a misregulated repair response. Cystic degeneration was more predominant in the PBS injected IVDs.

**Figure S4.** Degenerative features associated with different regions of annular lesions which have propagated from the outer AF through the mid and inner AF towards the contralateral AF. These features are shown diagrammatically in C. Cell clustering in the inner (A) and outer AF (B). Blood vessel ingrowth into the mid (D) and inner AF (E). H&E stained tissue sections.

**Figure S5.** Cellular morphology of the inner (A) and mid to outer AF (B) in normal nonoperated control (NOC) IVDs. Occasional doublet cells are observed in the transitional zone located between the mid and inner AF. Toluidine‐blue‐fast green stained tissue sections.

**Figure S6.** Degenerative propagation of the controlled outer annular lesions in toluidine blue‐fast green stained PBS injected IVDs. 1. Focal loss of proteoglycan staining associated with the outer lesion in the EA (A), LA (B, C, D) and EST treatment groups (E) in IVDs that received PBS carrier injections and no stem cells. 2. Bifurcation and 3. de‐lammelation of the lesion. The lesion was more extensive in the EST group and associated with lower proteoglycan levels and a decreased disc height, and 4. extended through to the contralateral AF. Toluidine blue‐fast green stained tissue vertical sections with portions of the superior and inferior vertebral bodies evident. A chondroid cell mass was also evident in (A) labeled with an asterisk.

**Figure S7.** Toluidine blue‐fast green stained vertical tissue sections from the early acute (EA), late acute (LA) and long‐term established (EST) disc degeneration treatment groups which were injected with MSCs or PBS carrier. Nonoperated IVDs are also shown for comparison, and a diagram depicting the location of the surgical annular lesion. Four MSC treated tissue sections are displayed from each of the MSC treatment groups. Lesion development is more advanced in the PBS carrier injected IVDs and disc heights also reduced.

**Figure S8.** Toluidine blue stained IVD tissue sections depicting cell clusters/cloning, chondroid cell nests and normal NP cell morphology. Examples of cell clustering associated with an annular lesion (A) and chondroid cell nests in the NP of a normal IVD (B, C) compared to cells in the central NP (D) and its margins with the AF (E). Occasional NP cells appear as doublets but are dissimilar to the chondroid cell nests contained in dense basophilic sacs which appear as larger groups of cells. These are similar to the cell clusters seen associated with annular lesions in degenerate IVDs however the chondroid cell nests are not associated with a fibrillar ECM like in A, D, E.

**Table S1.** Histopathological scoring of ovine IVDs

**Table S2.** qRTPCR primer details

## ENDNOTE

1 Further information on changes in Australian TGA guidelines on the use of stem cells are available from the following web‐sites. *Products regulated as biologicals.* //www.tga.gov.au/products-regulated-biologicals. *Regulatory framework for biologicals.* //www.tga.gov.au/regulatory-framework-biologicals. *Autologous human platelet‐rich plasma and conditioned serum* //www.tga.gov.au/autologoushuman-platelet-rich-plasma-and-conditioned-serum.

## References

[1] Shapiro I , Risbud MV . In: Shapiro IM, Risbud MV, eds. Ch. 1. The Intervertebral Disc: Molecular and Structural Studies of the Disc in Health and Disease. Springer‐Verlag; Vienna, Heidelberg London, NY 2014:3‐16.

[2] Little JP , Pearcy MJ , Tevelen G , Evans JH , Pettet G , Adam CJ . The mechanical response of the ovine lumbar anulus fibrosus to uniaxial, biaxial and shear loads. J Mech Behav Biomed Mater. 2010;3:146‐157. 10.1016/j.jmbbm.2009.09.002. 20129414

[3] Newell N , Little JP , Christou A , Adams MA , Adam CJ , Masouros SD . Biomechanics of the human intervertebral disc: a review of testing techniques and results. J Mech Behav Biomed Mater. 2017;69:420‐434. 10.1016/j.jmbbm.2017.01.037. 28262607

[4] Humzah MD , Soames RW . Human intervertebral disc: structure and function. Anat Rec. 1988;220:337‐356. 3289416 10.1002/ar.1092200402

[5] Melrose J , Roughley PJ . In: Shapiro IM, Risbud MV, eds. Ch. 4. The Intervertebral Disc. Molecular and Structural Studies of the Disc in Health and Disease. Springer‐Verlag; Vienna, Heidelberg London, NY 2014:53‐78.

[6] Roughley PJ , Melching LI , Heathfield TF , Pearce RH , Mort JS . The structure and degradation of aggrecan in human intervertebral disc. Eur Spine J. 2006;15(Suppl 3):S326‐S332. 10.1007/s00586-006-0127-7. 16736203 PMC2335376

[7] Sivan SS , Wachtel E , Roughley P . Structure, function, aging and turnover of aggrecan in the intervertebral disc. Biochim Biophys Acta. 2014;1840:3181‐3189. 10.1016/j.bbagen.2014.07.013. 25065289

[8] Roughley PJ . Biology of intervertebral disc aging and degeneration: involvement of the extracellular matrix. Spine (Phila Pa 1976). 2004;29:2691‐2699. 15564918 10.1097/01.brs.0000146101.53784.b1

[9] Roughley PJ , Alini M , Antoniou J . The role of proteoglycans in aging, degeneration and repair of the intervertebral disc. Biochem Soc Trans. 2002;30:869‐874. 12440935 10.1042/bst0300869

[10] Vernon‐Roberts B , Moore RJ , Fraser RD . The natural history of age‐related disc degeneration: the pathology and sequelae of tears. Spine (Phila Pa 1976). 2007;32:2797‐2804. 10.1097/BRS.0b013e31815b64d2. 18246000

[11] Vos T , Flaxman AD , Naghavi M , et al. Years lived with disability (YLDs) for 1160 sequelae of 289 diseases and injuries 1990‐2010: a systematic analysis for the global burden of disease study 2010. Lancet. 2012;380:2163‐2196. 10.1016/s0140-6736(12)61729-2. 23245607 PMC6350784

[12] Maniadakis N , Gray A . The economic burden of back pain in the UK. Pain. 2000;84:95‐103. 10601677 10.1016/S0304-3959(99)00187-6

[13] Walker BF , Muller R , Grant WD . Low back pain in Australian adults: the economic burden. Asia Pac J Public Health. 2003;15:79‐87. 15038680 10.1177/101053950301500202

[14] Jacobs JJ , Andersson G , Bell E‐J , et al. The Burden of Musculoskeletal Diseases in the United States. Vol. Chapter 9. American Academy of Surgeons; 6300 N. River Road Rosemont, IL, USA 2008.

[15] GBD 2015 Disease and Injury Incidence and Prevalence Collaborators . Global, regional, and national incidence, prevalence, and years lived with disability for 310 diseases and injuries, 1990–2015: a systematic analysis for the global burden of disease study 2015. Lancet. 2016;388:1545‐1602. 10.1016/s0140-6736(16)31678-6. 27733282 PMC5055577

[16] Ehrlich G , Khaltaev NG . WHO/NCD/NCM/CRA/99.1 In: Ehrlich GE, Khaltaev NG, eds. Chronic Respiratory Diseases and Arthritis Team. Vol 152 Geneva: World Health Organisation; 1999.

[17] Briggs AM , Buchbinder R . Back pain: a National Health Priority Area in Australia? Med J Aust. 2009;190:499‐502. 19413521 10.5694/j.1326-5377.2009.tb02527.x

[18] Spees JL , Lee RH , Gregory CA . Mechanisms of mesenchymal stem/stromal cell function. Stem Cell Res Ther. 2016;7:125 10.1186/s13287-016-0363-7. 27581859 PMC5007684

[19] Hang D , Li F , Che W , et al. One‐stage positron emission tomography and magnetic resonance imaging to assess mesenchymal stem cell survival in a canine model of intervertebral disc degeneration. Stem Cells Dev. 2017;26:1334‐1343. 10.1089/scd.2017.0103. 28665183

[20] Shi X , Liu J , Yang T , Zhang Y , Li T , Chen J . TLR2/NFkappaB signalling regulates endogenous IL‐6 release from marrow‐derived mesenchymal stromal cells to suppress the apoptosis of PC12 cells injured by oxygen and glucose deprivation. Mol Med Rep. 2016;13:5358‐5364. 10.3892/mmr.2016.5158. 27108485

[21] Song YS , Joo HW , Park IH , et al. Bone marrow mesenchymal stem cell‐derived vascular endothelial growth factor attenuates cardiac apoptosis via regulation of cardiac miRNA‐23a and miRNA‐92a in a rat model of myocardial infarction. PLoS One. 2017;12:e0179972. 10.1371/journal.pone.0179972. PMC5491110 28662151

[22] Bifari F , Lisi V , Mimiola E , Pasini A , Krampera M . Immune modulation by mesenchymal stem cells. Transfus Med Hemother. 2008;35:194‐204. 10.1159/000128968. 21547117 PMC3083287

[23] Bruno S , Deregibus MC , Camussi G . The secretome of mesenchymal stromal cells: role of extracellular vesicles in immunomodulation. Immunol Lett. 2015;168:154‐158. 10.1016/j.imlet.2015.06.007. 26086886

[24] Caplan AI , Sorrell JM . The MSC curtain that stops the immune system. Immunol Lett. 2015;168:136‐139. 10.1016/j.imlet.2015.06.005. 26079607

[25] Fibbe WE , Nauta AJ , Roelofs H . Modulation of immune responses by mesenchymal stem cells. Ann N Y Acad Sci. 2007;1106:272‐278. 10.1196/annals.1392.025. 17442776

[26] Rasmusson I . Immune modulation by mesenchymal stem cells. Exp Cell Res. 2006;312:2169‐2179. 10.1016/j.yexcr.2006.03.019. 16631737

[27] Caplan AI . Mesenchymal stem cells: time to change the name! Stem Cells Transl Med. 2017;6:1445‐1451. 10.1002/sctm.17-0051. 28452204 PMC5689741

[28] Melrose J . Strategies in regenerative medicine for intervertebral disc repair using mesenchymal stem cells and bioscaffolds. Regen Med. 2016;11:705‐724. 10.2217/rme-2016-0069. 27586197

[29] Melrose J . The promise of mesenchymal stem cells for intervertebral disc repair. J Stem Cell Res Ther. 2017;2:61‐64.

[30] Khan S , Mafi P , Mafi R , Khan W . A systematic review of mesenchymal stem cells in spinal cord injury, intervertebral disc repair and spinal fusion. Curr Stem Cell Res Ther. 2017;13:316‐323. 10.2174/1574888x11666170907120030. 28891440

[31] Anderson DG , Markova D , An HS , et al. Human umbilical cord blood‐derived mesenchymal stem cells in the cultured rabbit intervertebral disc: a novel cell source for disc repair. Am J Phys Med Rehabil. 2013;92:420‐429. 10.1097/PHM.0b013e31825f148a. 23598901 PMC4238943

[32] Feng G , Zhao X , Liu H , et al. Transplantation of mesenchymal stem cells and nucleus pulposus cells in a degenerative disc model in rabbits: a comparison of 2 cell types as potential candidates for disc regeneration. J Neurosurg Spine. 2011;14:322‐329. 10.3171/2010.11.spine10285. 21250814

[33] Liu K , Chen Z , Luo XW , et al. Determination of the potential of induced pluripotent stem cells to differentiate into mouse nucleus pulposus cells in vitro. Genet Mol Res. 2015;14:12394‐12405. 10.4238/2015.October.16.6. 26505389

[34] Freeman BJ , Kuliwaba JS , Jones CF , et al. Allogeneic mesenchymal precursor cells promote healing in Postero‐lateral annular lesions and improve indices of lumbar intervertebral disc degeneration in an ovine model. Spine (Phila Pa 1976). 2016;41:1331‐1339. 10.1097/brs.0000000000001528. 26913464

[35] Mwale F , Wang HT , Roughley P , Antoniou J , Haglund L . Link N and mesenchymal stem cells can induce regeneration of the early degenerate intervertebral disc. Tissue Eng Part A. 2014;20:2942–9. 10.1089/ten.TEA.2013.0749. PMC4229694 24786145

[36] Yang F , Leung VY , Luk KD , Chan D , Cheung KM . Mesenchymal stem cells arrest intervertebral disc degeneration through chondrocytic differentiation and stimulation of endogenous cells. Mol Ther. 2009;17:1959‐1966. 10.1038/mt.2009.146. 19584814 PMC2835041

[37] Orozco L , Soler R , Morera C , Alberca M , Sánchez A , García‐Sancho J . Intervertebral disc repair by autologous mesenchymal bone marrow cells: a pilot study. Transplantation. 2011;92:822‐828. 10.1097/TP.0b013e3182298a15. 21792091

[38] Oehme D , Goldschlager T , Rosenfeld JV , Ghosh P , Jenkin G . The role of stem cell therapies in degenerative lumbar spine disease: a review. Neurosurg Rev. 2015;38:429‐445. 10.1007/s10143-015-0621-7. 25749802

[39] Pennicooke B , Moriguchi Y , Hussain I , Bonssar LH , Härtl R. Biological treatment approaches for degenerative disc disease: a review of clinical trials and future directions. Cureus. 2016;8:e892. 10.7759/cureus.892. PMC5178982 28018762

[40] Coric D , Pettine K , Sumich A , Boltes MO . Prospective study of disc repair with allogeneic chondrocytes presented at the 2012 joint spine section meeting. J Neurosurg Spine. 2013;18:85‐95. 10.3171/2012.10.spine12512. 23140128

[41] Pettine KA , Murphy MB , Suzuki RK , Sand TT . Percutaneous injection of autologous bone marrow concentrate cells significantly reduces lumbar discogenic pain through 12 months. Stem Cells. 2015;33:146‐156. 10.1002/stem.1845. 25187512

[42] Mochida J , Sakai D , Nakamura Y , Watanabe T , Yamamoto Y , Kato S . Intervertebral disc repair with activated nucleus pulposus cell transplantation: a three‐year, prospective clinical study of its safety. Eur Cell Mater. 2015;29:202‐212; discussion 212. 25794529 10.22203/ecm.v029a15

[43] Elabd C , Centeno CJ , Schultz JR , Lutz G , Ichim T , Silva FJ . Intra‐discal injection of autologous, hypoxic cultured bone marrow‐derived mesenchymal stem cells in five patients with chronic lower back pain: a long‐term safety and feasibility study. J Transl Med. 2016;14:253 10.1186/s12967-016-1015-5. 27585696 PMC5009698

[44] Sakai D , Schol J . Cell therapy for intervertebral disc repair: clinical perspective. J Orthop Translat. 2017;9:8‐18. 10.1016/j.jot.2017.02.002. 29662795 PMC5822958

[45] Yim RL , Lee JT , Bow CH , et al. A systematic review of the safety and efficacy of mesenchymal stem cells for disc degeneration: insights and future directions for regenerative therapeutics. Stem Cells Dev. 2014;23:2553‐2567. 10.1089/scd.2014.0203. 25050446 PMC4201280

[46] Richardson SM , Kalamegam G , Pushparaj PN , et al. Mesenchymal stem cells in regenerative medicine: focus on articular cartilage and intervertebral disc regeneration. Methods. 2016;99:69‐80. 10.1016/j.ymeth.2015.09.015. 26384579

[47] Sakai D , Andersson GB . Stem cell therapy for intervertebral disc regeneration: obstacles and solutions. Nat Rev Rheumatol. 2015;11:243‐256. 10.1038/nrrheum.2015.13. 25708497

[48] Vadala G , Russo F , Ambrosio L , Papalia R , Denaro V . Mesenchymal stem cells for intervertebral disc regeneration. J Biol Regul Homeost Agents. 2016;30:173‐179. 28002916

[49] Wang HQ . Bring stem cell therapies to cure intervertebral disc degeneration to the forefront. Curr Stem Cell Res Ther. 2015;10:284. 25982533 10.2174/1574888x1004150513162605

[50] Wang LT , Ting CH , Yen ML , et al. Human mesenchymal stem cells (MSCs) for treatment towards immune‐ and inflammation‐mediated diseases: review of current clinical trials. J Biomed Sci. 2016;23:76 10.1186/s12929-016-0289-5. 27809910 PMC5095977

[51] Daly C , Ghosh P , Jenkin G , Oehme D , Goldschlager T . A review of animal models of intervertebral disc degeneration: pathophysiology, regeneration, and translation to the clinic. Biomed Res Int. 2016;2016:5952165‐5952114. 10.1155/2016/5952165. 27314030 PMC4893450

[52] Shu CC , Smith MM , Smith SM , et al. A histopathological scheme for the quantitative scoring of intervertebral disc degeneration and the therapeutic utility of adult mesenchymal stem cells for intervertebral disc regeneration. Int J Mol Sci. 2017;18:pii: E1049 10.3390/ijms18051049. PMC5454961 28498326

[53] Melrose J , Shu C , Young C , et al. Mechanical destabilization induced by controlled annular incision of the intervertebral disc dysregulates metalloproteinase expression and induces disc degeneration. Spine (Phila Pa 1976). 2012;37:18‐25. 10.1097/BRS.0b013e31820cd8d5. 22179320

[54] Shu C , Smith SM , Little CB , Melrose J . Use of FGF‐2 and FGF‐18 to direct bone marrow stromal stem cells to chondrogenic and osteogenic lineages. Future Sci OA. 2016;2:FSO142. 10.4155/fsoa-2016-0034. PMC5242207 28116125

[55] Andrade W , Seabrook TJ , Johnston MG , Hay JB . The use of the lipophilic fluorochrome CM‐DiI for tracking the migration of lymphocytes. J Immunol Methods. 1996;194:181‐189. 8765171 10.1016/0022-1759(96)00083-x

[56] Frobin W , Brinckmann P , Biggemann M , Tillotson M , Burton K . Precision measurement of disc height, vertebral height and sagittal plane displacement from lateral radiographic views of the lumbar spine. Clin Biomech (Bristol, Avon). 1997;12(Suppl 1):S1‐S63. 10.1016/s0268-0033(96)00067-8 11430783

[57] Clarke EC , Appleyard RC , Bilston LE . Immature sheep spines are more flexible than mature spines: an in vitro biomechanical study. Spine (Phila Pa 1976). 2007;32:2970‐2979. 10.1097/BRS.0b013e31815cde16. 18091489

[58] Farndale RW , Buttle DJ , Barrett AJ . Improved quantitation and discrimination of sulphated glycosaminoglycans by use of dimethylmethylene blue. Biochim Biophys Acta. 1986;883:173‐177. 3091074 10.1016/0304-4165(86)90306-5

[59] Stegemann H , Stalder K . Determination of hydroxyproline. Clin Chim Acta. 1967;18:267‐273. 4864804 10.1016/0009-8981(67)90167-2

[60] Osti OL , Vernon‐Roberts B , Fraser RD . 1990 Volvo award in experimental studies. Anulus tears and intervertebral disc degeneration An experimental study using an animal model. Spine. 1990;15:762‐767. 2237626 10.1097/00007632-199008010-00005

[61] Colloca CJ , Gunzburg R , Freeman BJ , Szpalski M , Afifi M , Moore RJ . Biomechancial quantification of pathologic manipulable spinal lesions: an in vivo ovine model of spondylolysis and intervertebral disc degeneration. J Manipulative Physiol Ther. 2012;35:354‐366. 10.1016/j.jmpt.2012.04.018. 22657392

[62] Colloca CJ , Keller TS , Harrison DE , et al. Spinal manipulation force and duration affect vertebral movement and neuromuscular responses. Clin Biomech (Bristol, Avon). 2006;21:254‐262. 10.1016/j.clinbiomech.2005.10.006. 16378668

[63] Colloca CJ , Keller TS , Moore RJ , Gunzburg R , Harrison DE . Intervertebral disc degeneration reduces vertebral motion responses. Spine (Phila Pa 1976). 2007;32:E544‐E550. 10.1097/BRS.0b013e318145ac39. 17762796

[64] Colloca CJ , Keller TS , Moore RJ , Gunzburg R , Harrison DE . Effects of disc degeneration on neurophysiological responses during dorsoventral mechanical excitation of the ovine lumbar spine. J Electromyogr Kinesiol. 2008;18:829‐837. 10.1016/j.jelekin.2007.02.017. 17468010

[65] Hodges PW , Galea MP , Holm S , Holm AK . Corticomotor excitability of back muscles is affected by intervertebral disc lesion in pigs. Eur J Neurosci. 2009;29:1490‐1500. 10.1111/j.1460-9568.2009.06670.x. 19519631

[66] Hodges PW , James G , Blomster L , et al. Multifidus muscle changes after Back injury are characterized by structural remodeling of muscle, adipose and connective tissue, but not muscle atrophy: molecular and morphological evidence. Spine (Phila Pa 1976). 2015;40:1057‐1071. 10.1097/brs.0000000000000972. 25943090

[67] Hodges P , Holm AK , Hansson T , Holm S . Rapid atrophy of the lumbar multifidus follows experimental disc or nerve root injury. Spine (Phila Pa 1976). 2006;31:2926‐2933. 10.1097/01.brs.0000248453.51165.0b. 17139223

[68] Hodges PW , James G , Blomster L , et al. Can proinflammatory cytokine gene expression explain multifidus muscle fiber changes after an intervertebral disc lesion? Spine (Phila Pa 1976). 2014;39:1010‐1017. 10.1097/brs.0000000000000318. 24718080

[69] Freeman MD , Woodham MA , Woodham AW . The role of the lumbar multifidus in chronic low back pain: a review. PM R. 2010;2:142‐146; quiz 141 p following 167. 10.1016/j.pmrj.2009.11.006. 20193941

[70] James G , Blomster L , Hall L , et al. Mesenchymal stem cell treatment of intervertebral disc lesion prevents fatty infiltration and fibrosis of the multifidus muscle, but not cytokine and muscle fiber changes. Spine (Phila Pa 1976). 2016;41:1208‐1217. 10.1097/brs.0000000000001669. 27135642

[71] James G , Sluka KA , Blomster L , et al. Macrophage polarization contributes to local inflammation and structural change in the multifidus muscle after intervertebral disc injury. Eur Spine J. 2018;27:1744‐1756. 10.1007/s00586-018-5652-7. 29948327

[72] Schollum ML , Appleyard RC , Little CB , Melrose J . A detailed microscopic examination of alterations in normal anular structure induced by mechanical destabilization in an ovine model of disc degeneration. Spine (Phila Pa 1976). 2010;35:1965‐1973. 10.1097/BRS.0b013e3181e0f085. 20959777

[73] Osti OL , Vernon‐Roberts B , Moore R , Fraser RD . Annular tears and disc degeneration in the lumbar spine. A post‐mortem study of 135 discs. J Bone Joint Surg Br. 1992;74:678‐682. 1388173 10.1302/0301-620X.74B5.1388173

[74] Melrose J , Smith SM , Little CB , Moore RJ , Vernon‐Roberts B , Fraser RD . Recent advances in annular pathobiology provide insights into rim‐lesion mediated intervertebral disc degeneration and potential new approaches to annular repair strategies. Eur Spine J. 2008;17:1131‐1148. 10.1007/s00586-008-0712-z. 18584218 PMC2527413

[75] Melrose J , Ghosh P , Taylor TKF , et al. A longitudinal study of the matrix changes induced in the intervertebral disc by surgical damage to the annulus fibrosus. J Orthop Res. 1992;10:665‐676. 1500980 10.1002/jor.1100100509

[76] Moore RJ , Osti OL , Vernon‐Roberts B , Fraser RD . Changes in endplate vascularity after an outer anulus tear in the sheep. Spine. 1992;17:874‐878. 1523489 10.1097/00007632-199208000-00003

[77] Moore RJ , Vernon‐Roberts B , Osti OL , Fraser RD . Remodeling of vertebral bone after outer anular injury in sheep. Spine. 1996;21:936‐940. 8726196 10.1097/00007632-199604150-00006

[78] Moore RJ , Crotti TN , Osti OL , Fraser RD , Vernon‐Roberts B . Osteoarthrosis of the facet joints resulting from anular rim lesions in sheep lumbar discs. Spine. 1999;24:519‐525. 10101813 10.1097/00007632-199903150-00003

[79] Melrose J , Roberts S , Smith S , Menage J , Ghosh P . Increased nerve and blood vessel ingrowth associated with proteoglycan depletion in an ovine anular lesion model of experimental disc degeneration. Spine. 2002;27:1278‐1285. 12065974 10.1097/00007632-200206150-00007

[80] Melrose J , Smith S , Little CB , Kitson J , Hwa SY , Ghosh P . Spatial and temporal localization of transforming growth factor‐beta, fibroblast growth factor‐2, and osteonectin, and identification of cells expressing alpha‐smooth muscle actin in the injured anulus fibrosus: implications for extracellular matrix repair. Spine. 2002;27:1756‐1764. 12195068 10.1097/00007632-200208150-00014

[81] Alini M , Eisenstein SM , Ito K , et al. Are animal models useful for studying human disc disorders/degeneration? Eur Spine J. 2008;17:2‐19. 17632738 10.1007/s00586-007-0414-y PMC2365516

[82] Liu S , Jiang L , Li H , et al. Mesenchymal stem cells prevent hypertrophic scar formation via inflammatory regulation when undergoing apoptosis. J Invest Dermatol. 2014;134:2648‐2657. 10.1038/jid.2014.169. 24714203

[83] Reinders ME , de Fijter JW , Rabelink TJ . Mesenchymal stromal cells to prevent fibrosis in kidney transplantation. Curr Opin Organ Transplant. 2014;19:54‐59. 10.1097/mot.0000000000000032. 24275894

[84] Ueno T , Nakashima A , Doi S , et al. Mesenchymal stem cells ameliorate experimental peritoneal fibrosis by suppressing inflammation and inhibiting TGF‐beta1 signaling. Kidney Int. 2013;84:297‐307. 10.1038/ki.2013.81. 23486522 PMC3731556

[85] Wu Y , Peng Y , Gao D , et al. Mesenchymal stem cells suppress fibroblast proliferation and reduce skin fibrosis through a TGF‐beta3‐dependent activation. Int J Low Extrem Wounds. 2015;14:50‐62. 10.1177/1534734614568373. 25858630

[86] Leung VY , Aladin DM , Lv F , et al. Mesenchymal stem cells reduce intervertebral disc fibrosis and facilitate repair. Stem Cells. 2014;32:2164‐2177. 10.1002/stem.1717. 24737495

[87] Kizil C , Kyritsis N , Brand M . Effects of inflammation on stem cells: together they strive? EMBO Rep. 2015;16:416‐426. 10.15252/embr.201439702. 25739812 PMC4388609

[88] Pers YM , Ruiz M , Noel D , Jorgensen C . Mesenchymal stem cells for the management of inflammation in osteoarthritis: state of the art and perspectives. Osteoarthr Cartil. 2015;23:2027‐2035. 10.1016/j.joca.2015.07.004. 26521749

[89] Murphy MB , Moncivais K , Caplan AI . Mesenchymal stem cells: environmentally responsive therapeutics for regenerative medicine. Exp Mol Med. 2013;45:e54. 10.1038/emm.2013.94. PMC3849579 24232253

[90] de Windt TS , Saris DB , Slaper‐Cortenbach IC , et al. Direct cell‐cell contact with chondrocytes is a key mechanism in multipotent mesenchymal stromal cell‐mediated chondrogenesis. Tissue Eng Part A. 2015;21:2536‐2547. 10.1089/ten.TEA.2014.0673. 26166387

[91] English K , Ryan JM , Tobin L , Murphy MJ , Barry FP , Mahon BP . Cell contact, prostaglandin E(2) and transforming growth factor beta 1 play non‐redundant roles in human mesenchymal stem cell induction of CD4+CD25(high) forkhead box P3+ regulatory T cells. Clin Exp Immunol. 2009;156:149‐160. 10.1111/j.1365-2249.2009.03874.x. 19210524 PMC2673753

[92] Meirelles Lda S , Fontes AM , Covas DT , Caplan AI . Mechanisms involved in the therapeutic properties of mesenchymal stem cells. Cytokine Growth Factor Rev. 2009;20:419‐427. 10.1016/j.cytogfr.2009.10.002. 19926330

[93] Chen L , Tredget EE , Wu PY , Wu Y . Paracrine factors of mesenchymal stem cells recruit macrophages and endothelial lineage cells and enhance wound healing. PLoS One. 2008;3:e1886. 10.1371/journal.pone.0001886. PMC2270908 18382669

[94] Chen YT , Sun CK , Lin YC , et al. Adipose‐derived mesenchymal stem cell protects kidneys against ischemia‐reperfusion injury through suppressing oxidative stress and inflammatory reaction. J Transl Med. 2011;9:51 10.1186/1479-5876-9-51. 21545725 PMC3112438

[95] Wu Y , Huang S , Enhe J , et al. Bone marrow‐derived mesenchymal stem cell attenuates skin fibrosis development in mice. Int Wound J. 2014;11:701‐710. 10.1111/iwj.12034. 23409729 PMC7950597

[96] Squillaro T , Peluso G , Galderisi U . Clinical trials with mesenchymal stem cells: an update. Cell Transplant. 2016;25:829‐848. 10.3727/096368915x689622. 26423725

[97] Okazaki S , Hisha H , Mizokami T , et al. Successful acceptance of adult liver allografts by intra‐bone marrow‐bone marrow transplantation. Stem Cells Dev. 2008;17:629‐639. 10.1089/scd.2007.0218. 18537462

[98] Zappia E , Casazza S , Pedemonte E , et al. Mesenchymal stem cells ameliorate experimental autoimmune encephalomyelitis inducing T‐cell anergy. Blood. 2005;106:1755‐1761. 10.1182/blood-2005-04-1496. 15905186

[99] Rissanen PM . Comparison of pathologic changes in intervertebral discs and interspinous ligaments of the lower part of the lumbar spine in the light of autopsy findings. Acta Orthop Scand. 1964;34:54‐65. 14124650 10.3109/17453676408989304

[100] Konar M , Lang J , Fluhmann G , Forterre F . Ventral intraspinal cysts associated with the intervertebral disc: magnetic resonance imaging observations in seven dogs. Vet Surg. 2008;37:94‐101. 10.1111/j.1532-950X.2007.00353.x. 18199062

[101] Hansen HJ . A pathologic‐anatomical study on disc degeneration in dog, with special reference to the so‐called enchondrosis intervertebralis. Acta Orthop Scand Suppl. 1952;11:1‐117. 14923291 10.3109/ort.1952.23.suppl-11.01

[102] Hansen T , Smolders LA , Tryfonidou MA , et al. The myth of fibroid degeneration in the canine intervertebral disc: a histopathological comparison of intervertebral disc degeneration in Chondrodystrophic and Nonchondrodystrophic dogs. Vet Pathol. 2017;54:945‐952. 10.1177/0300985817726834. 28847244

[103] Smolders LA , Bergknut N , Grinwis GCM , et al. Intervertebral disc degeneration in the dog. Part 2: chondrodystrophic and non‐chondrodystrophic breeds. Vet J. 2013;195:292‐299. 10.1016/j.tvjl.2012.10.011. 23154070

[104] Green PW , Fox RR , Sokoloff L . Spontaneous degenerative spinal disease in the laboratory rabbit. J Orthop Res. 1984;2:161‐168. 10.1002/jor.1100020207. 6491810

[105] Rohdin C , Jeserevic J , Viitmaa R , Cizinauskas S . Prevalence of radiographic detectable intervertebral disc calcifications in dachshunds surgically treated for disc extrusion. Acta Vet Scand. 2010;52:24 10.1186/1751-0147-52-24. 20398282 PMC2873269

[106] Melrose J , Burkhardt D , Taylor TKF , et al. Calcification in the ovine intervertebral disc: a model of hydroxyapatite deposition disease. Eur Spine J. 2009;18:479‐489. 10.1007/s00586-008-0871-y. 19165512 PMC2899463

[107] Braund KG , Ghosh P , Taylor TK , Larsen LH . Morphological studies of the canine intervertebral disc. The assignment of the beagle to the achondroplastic classification. Res Vet Sci. 1975;19:167‐172. 1166121

[108] Mallett J . Famous sheep breeds: the Merino. J Dept Agriclture Western Australia. 1, 33‐39. 1960;1, https://researchlibrary.agric.wa.gov.au/journal_agriculture4/vol1/iss1/6.

[109] Bogduk N . The lumbar disc and low back pain. Neurosurg Clin N Am. 1991;2:791‐806. 1821758

[110] Hartvigsen J , Christensen K , Frederiksen H . Back pain remains a common symptom in old age. A population‐based study of 4486 Danish twins aged 70‐102. Eur Spine J. 2003;12:528‐534. 10.1007/s00586-003-0542-y. 12748896 PMC3468008

[111] Hartvigsen J , Hancock MJ , Kongsted A , et al. What low back pain is and why we need to pay attention. Lancet. 2018;391:2356‐2367. 10.1016/s0140-6736(18)30480-x. 29573870

[112] Hoy D , Bain C , Williams G , et al. A systematic review of the global prevalence of low back pain. Arthritis Rheum. 2012;64:2028‐2037. 10.1002/art.34347. 22231424

[113] Kamper SJ , Henschke N , Hestbaek L , Dunn KM , Williams CM . Musculoskeletal pain in children and adolescents. Braz J Phys Ther. 2016;20:275‐284. 10.1590/bjpt-rbf.2014.0149. 27437719 PMC4946844

[114] Fagan A , Moore R , Vernon Roberts B , Blumbergs P , Fraser R . ISSLS prize winner: the innervation of the intervertebral disc: a quantitative analysis. Spine (Phila Pa 1976). 2003;28:2570‐2576. 10.1097/01.brs.0000096942.29660.b1. 14652473

[115] Freemont AJ , Peacock TE , Goupille P , Hoyland JA , O'Brien J , Jayson MIV . Nerve ingrowth into diseased intervertebral disc in chronic back pain. Lancet. 1997;350:178‐181. 9250186 10.1016/s0140-6736(97)02135-1

[116] de Chaumont F , Coura RDS , Serreau P , et al. Computerized video analysis of social interactions in mice. Nat Methods. 2012;9:410‐417. 10.1038/nmeth.1924. 22388289

[117] Gomez‐Marin A , Partoune N , Stephens GJ , Louis M , Brembs B . Automated tracking of animal posture and movement during exploration and sensory orientation behaviors. PLoS One. 2012;7:e41642. 10.1371/journal.pone.0041642. PMC3415430 22912674

[118] Ohayon S , Avni O , Taylor AL , Perona P , Roian Egnor SE . Automated multi‐day tracking of marked mice for the analysis of social behaviour. J Neurosci Methods. 2013;219:10‐19. 10.1016/j.jneumeth.2013.05.013. 23810825 PMC3762481

[119] Stern U , He R , Yang CH . Analyzing animal behavior via classifying each video frame using convolutional neural networks. Sci Rep. 2015;5:14351 10.1038/srep14351. 26394695 PMC4585819

[120] Millecamps M , Czerminski JT , Mathieu AP , Stone LS . Behavioral signs of axial low back pain and motor impairment correlate with the severity of intervertebral disc degeneration in a mouse model. Spine J. 2015;15:2524‐2537. 10.1016/j.spinee.2015.08.055. 26334234

[121] Miyagi M , Ishikawa T , Kamoda H , et al. Assessment of pain behavior in a rat model of intervertebral disc injury using the CatWalk gait analysis system. Spine (Phila Pa 1976). 2013;38:1459‐1465. 10.1097/BRS.0b013e318299536a. 23649215

[122] Miyagi M , Millecamps M , Danco AT , Ohtori S , Takahashi K , Stone LS . ISSLS prize winner: increased innervation and sensory nervous system plasticity in a mouse model of low back pain due to intervertebral disc degeneration. Spine (Phila Pa 1976). 2014;39:1345‐1354. 10.1097/brs.0000000000000334. 24718079

[123] Zhou Y , Hu X , Zheng X , et al. Differentiation potential of mesenchymal stem cells derived from adipose tissue vs bone marrow toward annulus Fibrosus cells in vitro. Curr Stem Cell Res Ther. 2017;12:432‐439. 10.2174/1574888x12666170214093955. 28201959

[124] Xu X , Hu J , Lu H . Histological observation of a gelatin sponge transplant loaded with bone marrow‐derived mesenchymal stem cells combined with platelet‐rich plasma in repairing an annulus defect. PLoS One. 2017;12:e0171500. 10.1371/journal.pone.0171500. PMC5298264 28178294

[125] Li X , Zhang Y , Song B , et al. Experimental application of bone marrow mesenchymal stem cells for the repair of intervertebral disc annulus Fibrosus. Med Sci Monit. 2016;22:4426‐4430. 27857031 10.12659/MSM.898062 PMC5124432

[126] Pirvu T , Blanquer SBG , Benneker LM , et al. A combined biomaterial and cellular approach for annulus fibrosus rupture repair. Biomaterials. 2015;42:11‐19. 10.1016/j.biomaterials.2014.11.049. 25542789

[127] Farrugia BL , Lord MS , Whitelock JM , Melrose J . Harnessing chondroitin sulphate in composite scaffolds to direct progenitor and stem cell function for tissue repair. Biomater Sci. 2018;6:947‐957. 10.1039/c7bm01158j. 29560990

[128] Rnjak‐Kovacina J , Tang F , Whitelock JM , Lord MS . Glycosaminoglycan and proteoglycan‐based biomaterials: current trends and future perspectives. Adv Healthc Mater. 2018;7:e1701042. 10.1002/adhm.201701042. 29210510

[129] Sharifi S , van Kooten TG , Kranenburg HJC , et al. An annulus fibrosus closure device based on a biodegradable shape‐memory polymer network. Biomaterials. 2013;34:8105‐8113. 10.1016/j.biomaterials.2013.07.061. 23932501

[130] Sharifi S , Kranenburg HJC , Meij BP . Photo‐crosslinkable poly(trimethylene carbonate)‐based macromers for closure of ruptured intervertebral discs. Macromol Symp. 2011;309‐310:100‐110.

[131] Graham LD , Danon SJ , Johnson G , et al. Biocompatibility and modification of the protein‐based adhesive secreted by the Australian frog Notaden bennetti. J Biomed Mater Res A. 2010;93:429‐441. 10.1002/jbm.a.32559. 19569213

[132] Graham LD , Glattauer V , Huson MG , et al. Characterization of a protein‐based adhesive elastomer secreted by the Australian frog Notaden bennetti. Biomacromolecules. 2005;6:3300‐3312. 10.1021/bm050335e. 16283759

[133] Graham LD , Glattauer V , Li D , Tyler MJ , Ramshaw JA . The adhesive skin exudate of Notaden bennetti frogs (Anura: Limnodynastidae) has similarities to the prey capture glue of Euperipatoides sp. velvet worms (Onychophora: Peripatopsidae). Comp Biochem Physiol B Biochem Mol Biol. 2013;165:250‐259. 10.1016/j.cbpb.2013.04.008. 23665109

[134] Millar NL , Bradley TA , Walsh NA , Appleyard RC , Tyler MJ , Murrell GAC . Frog glue enhances rotator cuff repair in a laboratory cadaveric model. J Shoulder Elbow Surg. 2009;18:639‐645. 10.1016/j.jse.2008.12.007. 19250843

[135] Nowak R . Frog glue repairs damaged cartilage. New Scientist. 2004.

[136] Szomor Z , Murrell GAC , Appleyard RC . Use of Notaden benetti Frog‐glue for Meniscal Repair. Tech Knee Surg. 2008;7:261‐265.

[137] Bochynska AI , Hannink G , Grijpma DW , Buma P . Tissue adhesives for meniscus tear repair: an overview of current advances and prospects for future clinical solutions. J Mater Sci Mater Med. 2016;27:85 10.1007/s10856-016-5694-5. 26970767 PMC4789195

[138] Bochynska AI , Hannink G , Janssen D , Buma P , Grijpma DW . Development of a fast curing tissue adhesive for meniscus tear repair. J Mater Sci Mater Med. 2017;28:1 10.1007/s10856-016-5790-6. 27866344 PMC5116306

[139] Bochynska AI , Van Tienen TG , Hannink G , Buma P , Grijpma DW . Development of biodegradable hyper‐branched tissue adhesives for the repair of meniscus tears. Acta Biomater. 2016;32:1‐9. 10.1016/j.actbio.2015.12.018. 26689469

[140] Brubaker CE , Messersmith PB . The present and future of biologically inspired adhesive interfaces and materials. Langmuir. 2012;28:2200‐2205. 10.1021/la300044v. 22224862

[141] Shao H , Bachus KN , Stewart RJ . A water‐borne adhesive modeled after the sandcastle glue of *P. californica* . Macromol Biosci. 2009;9:464‐471. 10.1002/mabi.200800252. 19040222 PMC2848666

[142] Liang L , Li X , Li D , et al. The characteristics of stem cells in human degenerative intervertebral disc. Medicine (Baltimore). 2017;96:e7178. 10.1097/md.0000000000007178. PMC5484206 28640098

[143] Liu C , Guo Q , Li J , et al. Identification of rabbit annulus fibrosus‐derived stem cells. PLoS One. 2014;9:e108239. 10.1371/journal.pone.0108239. PMC4178129 25259600

[144] Liu S , Liang H , Lee SM , Li Z , Zhang J , Fei Q . Isolation and identification of stem cells from degenerated human intervertebral discs and their migration characteristics. Acta Biochim Biophys Sin (Shanghai). 2017;49:101‐109. 10.1093/abbs/gmw121. 28172101

[145] Sang C , Cao X , Chen F , Yang X , Zhang Y . Differential characterization of two kinds of stem cells isolated from rabbit nucleus pulposus and annulus Fibrosus. Stem Cells Int. annulus Fibrosus. Stem Cells Int. 2016;14:8283257 10.1155/2016/8283257. PMC5040834 27703485

[146] Brown S , Matta A , Erwin M , et al. Cell clusters are indicative of stem cell activity in the degenerate intervertebral disc: can their properties be manipulated to improve intrinsic repair of the disc? Stem Cells Dev. 2018;27:147‐165. 10.1089/scd.2017.0213. 29241405

[147] Fuller ES , Shu C , Smith MM , Little CB , Melrose J . Hyaluronan oligosaccharides stimulate mmp and anabolic gene expression in‐vitro by intervertebral disc cells and annular repair in‐vivo. J Tissue Eng Regen Med. 2016;12:e216‐e226. 10.1002/term.2319. 27689852

[148] Cho H , Park SH , Park K , et al. Construction of a tissue‐engineered annulus fibrosus. Artif Organs. 2013;37:E131‐E138. 10.1111/aor.12066. 23621741

[149] Nerurkar NL , Mauck RL , Elliott DM . Modeling interlamellar interactions in angle‐ply biologic laminates for annulus fibrosus tissue engineering. Biomech Model Mechanobiol. 2011;10:973‐984. 10.1007/s10237-011-0288-0. 21287395 PMC3513349

