# Supplementary figures and images for: Efficacy of administered mesenchymal stem cells in the initiation and co‐ordination of repair processes by resident disc cells in an ovine (Ovis aries) large destabilizing lesion model of experimental disc degeneration
Source: JOR Spine. 2018 Oct 10;1(4):e1037. doi: 10.1002/jsp2.1037 (PMC6686814; doi:10.1002/jsp2.1037)

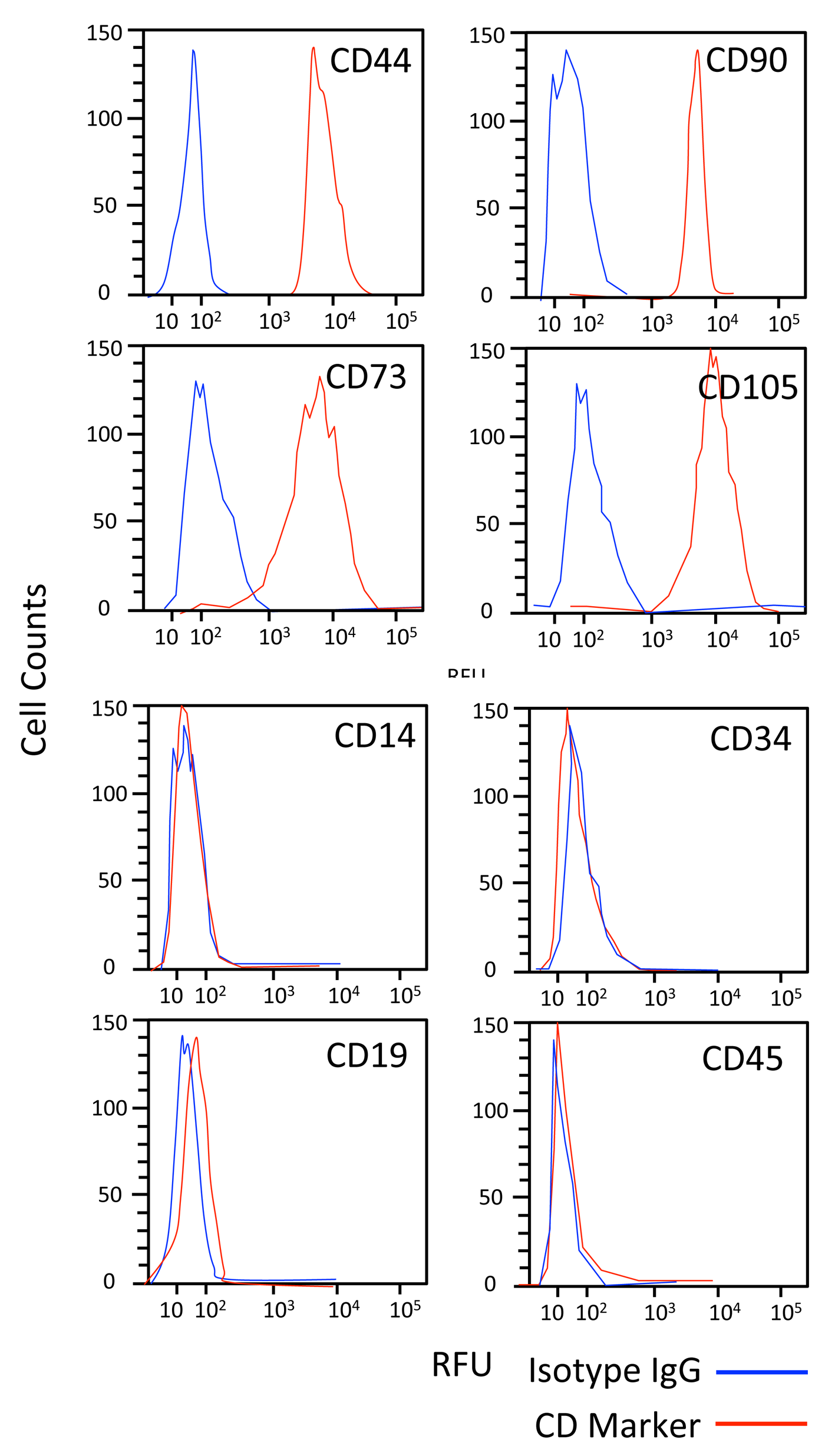

Supplement: Supplementary file 1 — Figure S1. Identification of CD34, CD45, CD90 and CD 105 expression by ovine bone marrow stromal stem cells and no staining for CD14, CD19, CD73 by flow cytometry. [file JSP2-1-e1037-s001.tif]

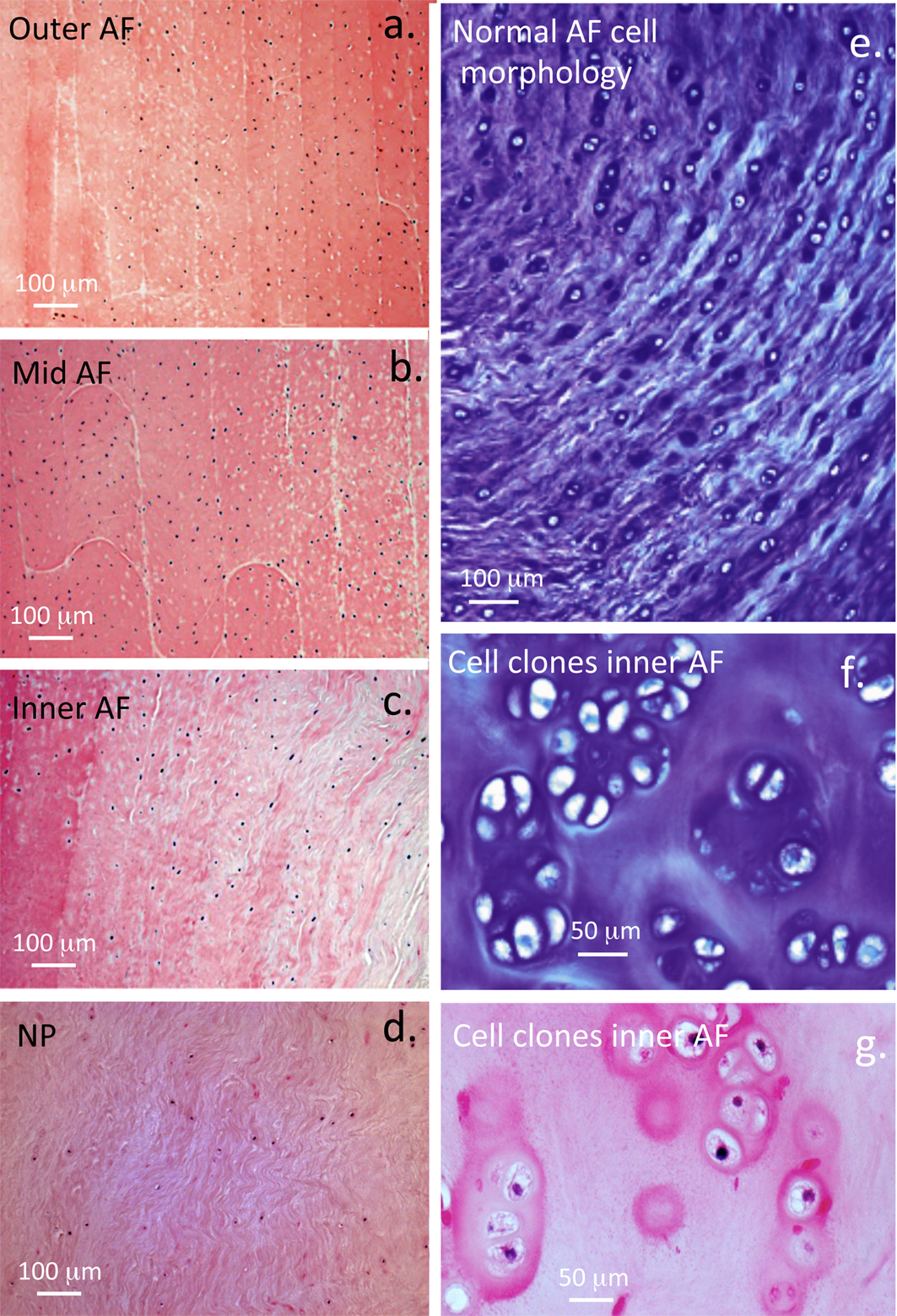

Supplement: Supplementary file 2 — Figure S2. Cellular morphology in the normal annulus and the dissimilar cell clusters observed in the mid and inner AF associated with annular lesion development in degenerate IVDs. H&E and toluidine blue‐fast green stained tissue sections. [file JSP2-1-e1037-s002.tif]

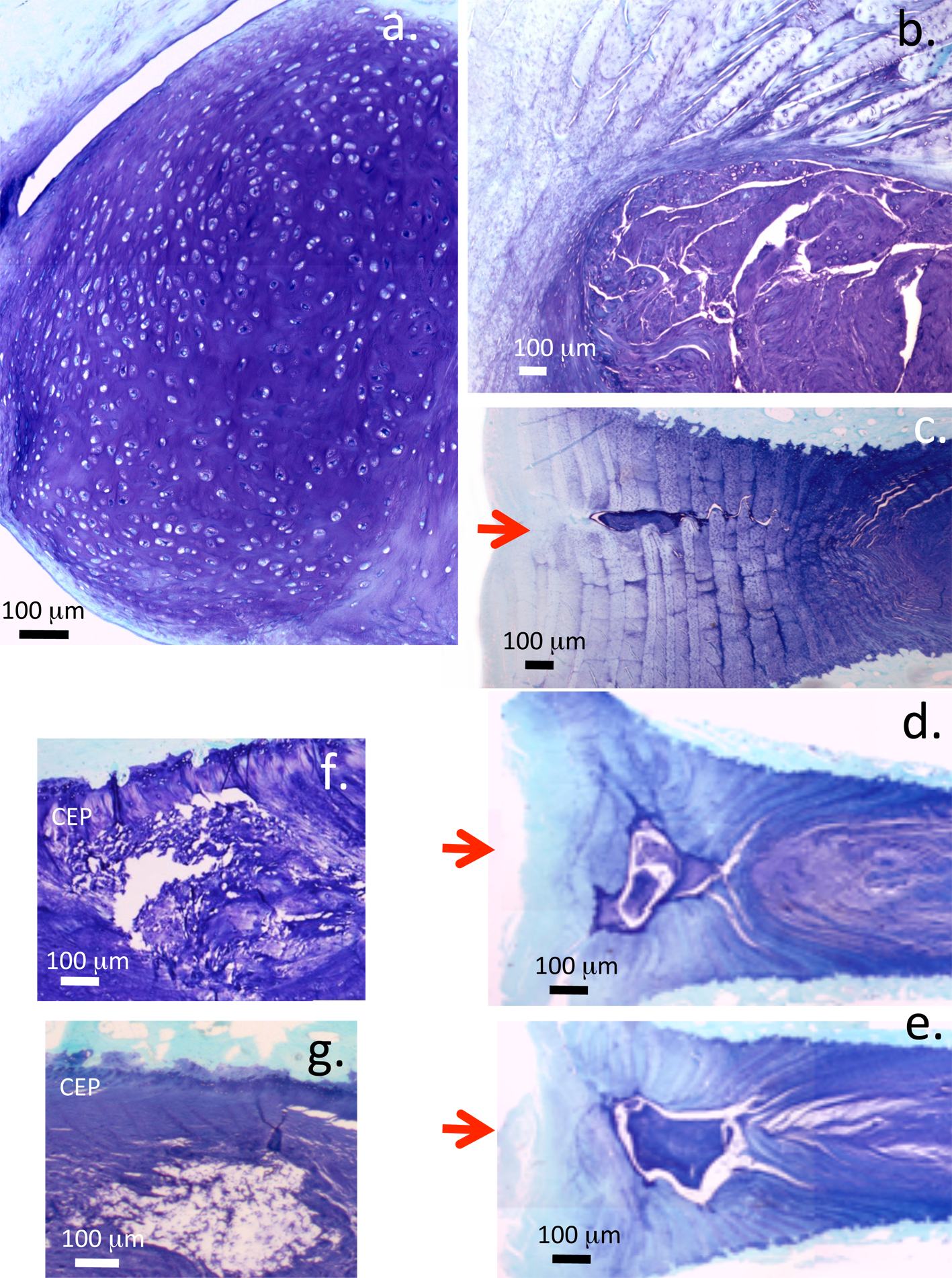

Supplement: Supplementary file 3 — Figure S3. Chondroid metaplasia (A‐E) and cystic degeneration (F, G) evident in lesion containing IVDs treated with MSCs. In some cases chondroid tissue along the tract of the annular lesion is integrated with the annular lamellae and appears to contribute to repair of the lesion (B, C) while in other samples this chondroid tissue occurs as an isolated tissue mass (D, E). Cells in the chondroid tissue have a typical rounded chondrocytic morphology surrounded by a proteoglycan rich cartilaginous ECM (A). Cyst formation in degenerate IVDs not treated with MSCs, typically occurring adjacent to the CEP (F, G). Toluidine blue‐fast green stained tissue sections. Tissue sections from EST MSC treatment group (A‐C), EA (D) and LA MSC treatment groups (E). Chondroid metaplasia only occurred in the MSC treated IVDs, this may represent a misregulated repair response. Cystic degeneration was more predominant in the PBS injected IVDs. [file JSP2-1-e1037-s003.tif]

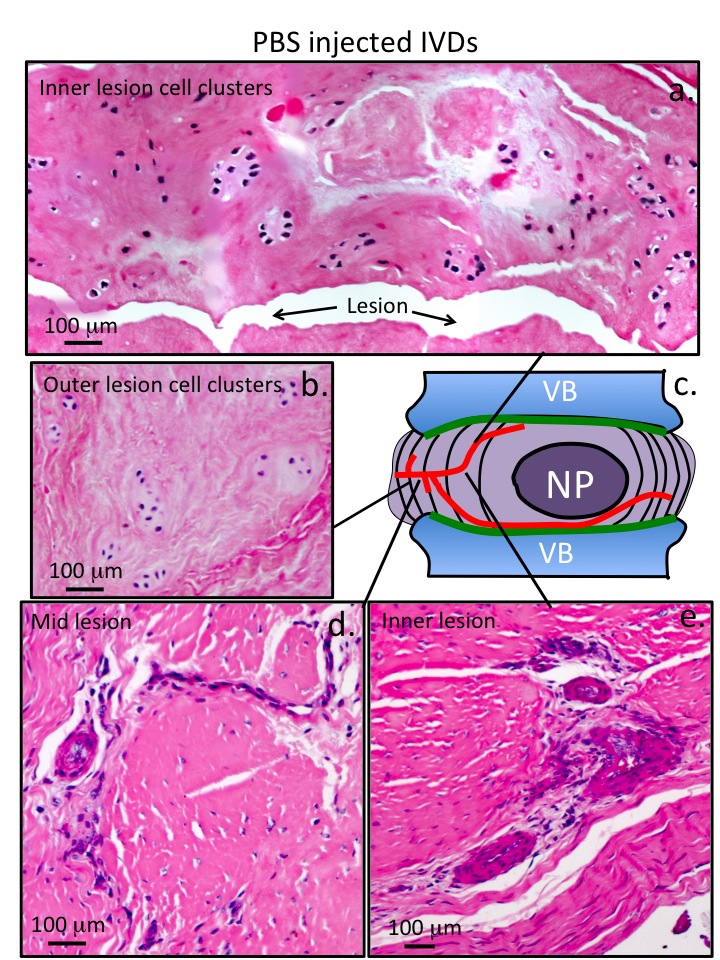

Supplement: Supplementary file 4 — Figure S4. Degenerative features associated with different regions of annular lesions which have propagated from the outer AF through the mid and inner AF towards the contralateral AF. These features are shown diagrammatically in C. Cell clustering in the inner (A) and outer AF (B). Blood vessel ingrowth into the mid (D) and inner AF (E). H&E stained tissue sections. [file JSP2-1-e1037-s004.jpg]

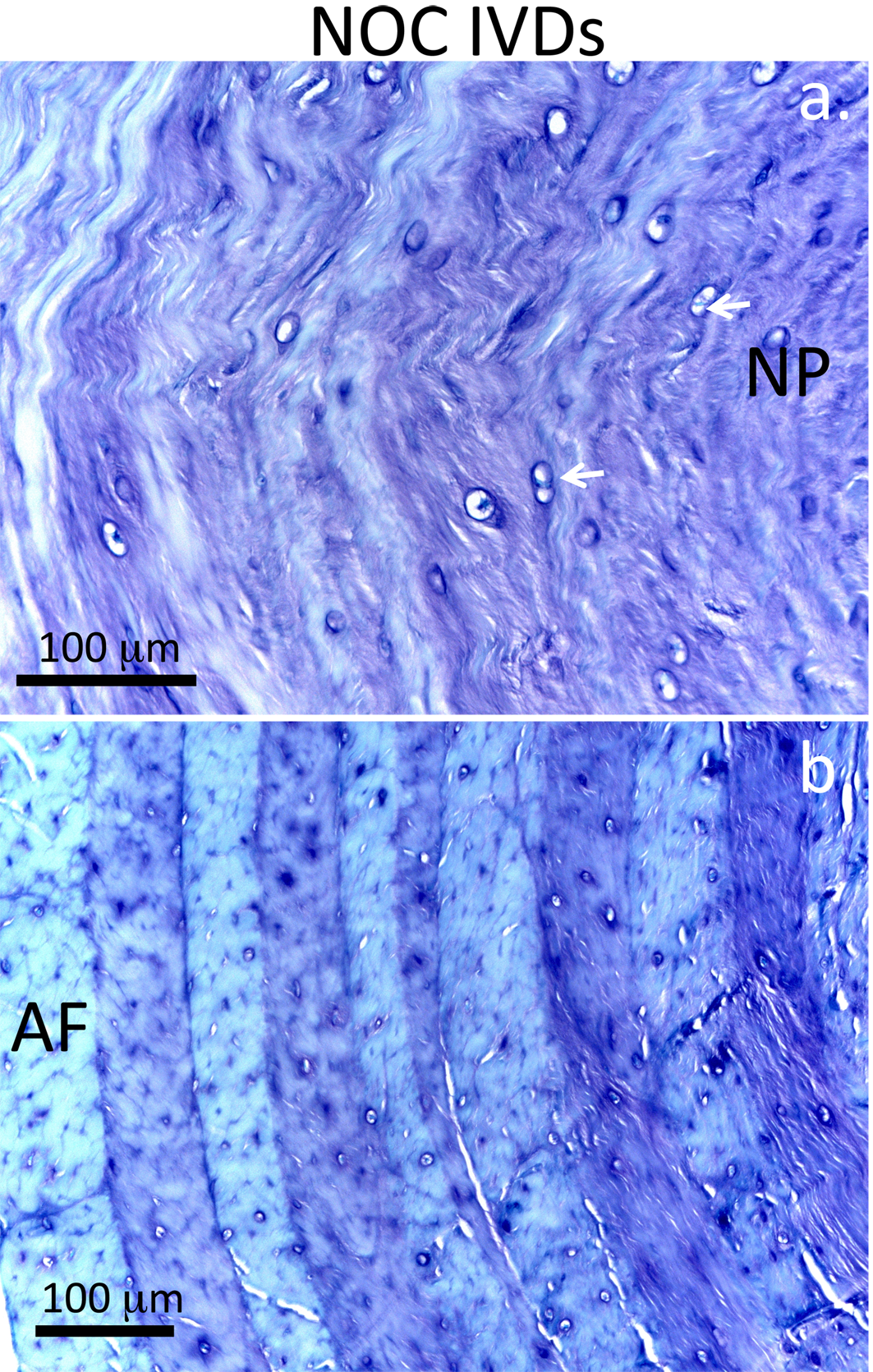

Supplement: Supplementary file 5 — Figure S5. Cellular morphology of the inner (A) and mid to outer AF (B) in normal nonoperated control (NOC) IVDs. Occasional doublet cells are observed in the transitional zone located between the mid and inner AF. Toluidine‐blue‐fast green stained tissue sections. [file JSP2-1-e1037-s005.tif]

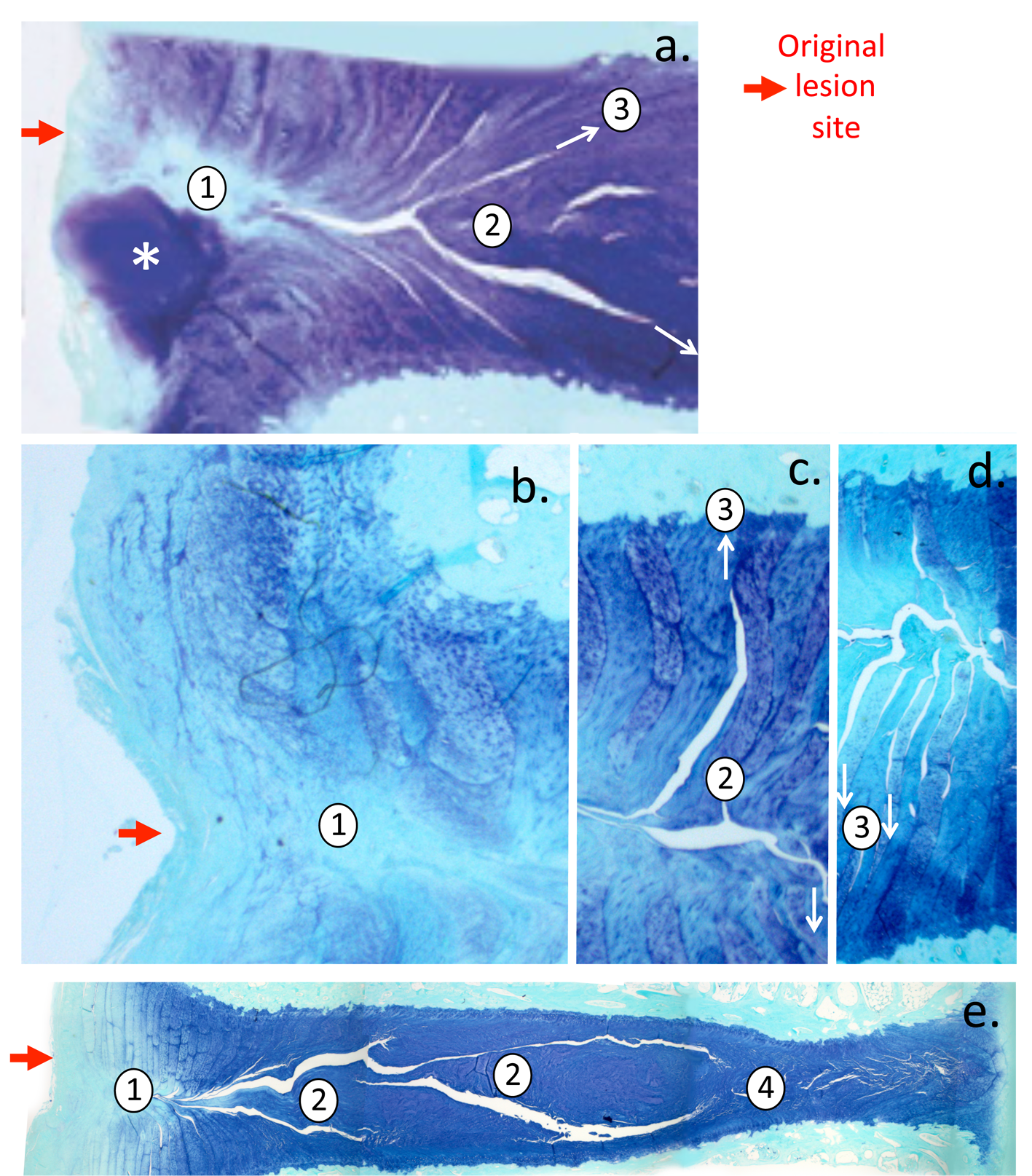

Supplement: Supplementary file 6 — Figure S6. Degenerative propagation of the controlled outer annular lesions in toluidine blue‐fast green stained PBS injected IVDs. 1. Focal loss of proteoglycan staining associated with the outer lesion in the EA (A), LA (B, C, D) and EST treatment groups (E) in IVDs that received PBS carrier injections and no stem cells. 2. Bifurcation and 3. de‐lammelation of the lesion. The lesion was more extensive in the EST group and associated with lower proteoglycan levels and a decreased disc height, and 4. extended through to the contralateral AF. Toluidine blue‐fast green stained tissue vertical sections with portions of the superior and inferior vertebral bodies evident. A chondroid cell mass was also evident in (A) labeled with an asterisk. [file JSP2-1-e1037-s006.tif]

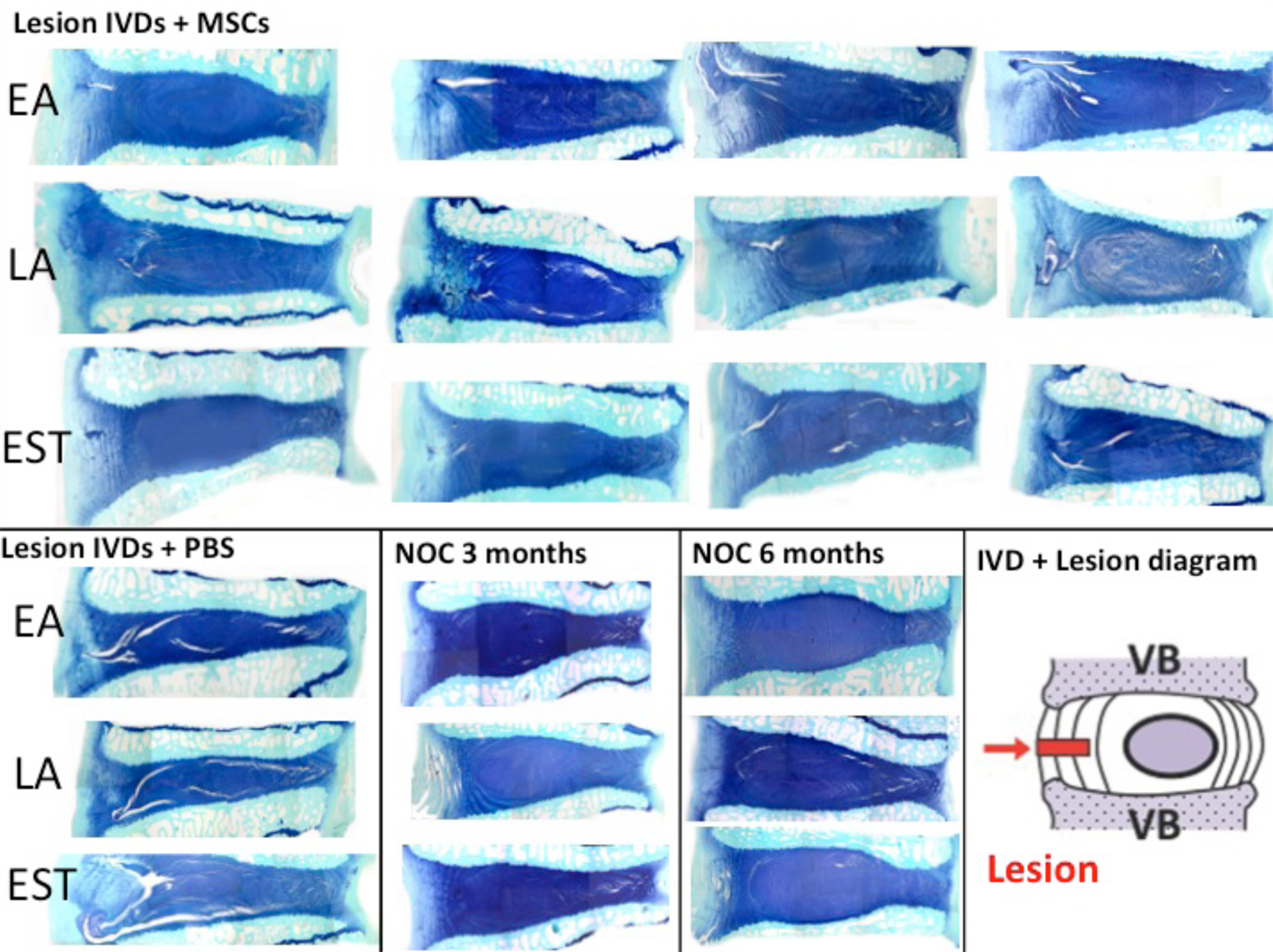

Supplement: Supplementary file 7 — Figure S7. Toluidine blue‐fast green stained vertical tissue sections from the early acute (EA), late acute (LA) and long‐term established (EST) disc degeneration treatment groups which were injected with MSCs or PBS carrier. Nonoperated IVDs are also shown for comparison, and a diagram depicting the location of the surgical annular lesion. Four MSC treated tissue sections are displayed from each of the MSC treatment groups. Lesion development is more advanced in the PBS carrier injected IVDs and disc heights also reduced. [file JSP2-1-e1037-s007.tif]

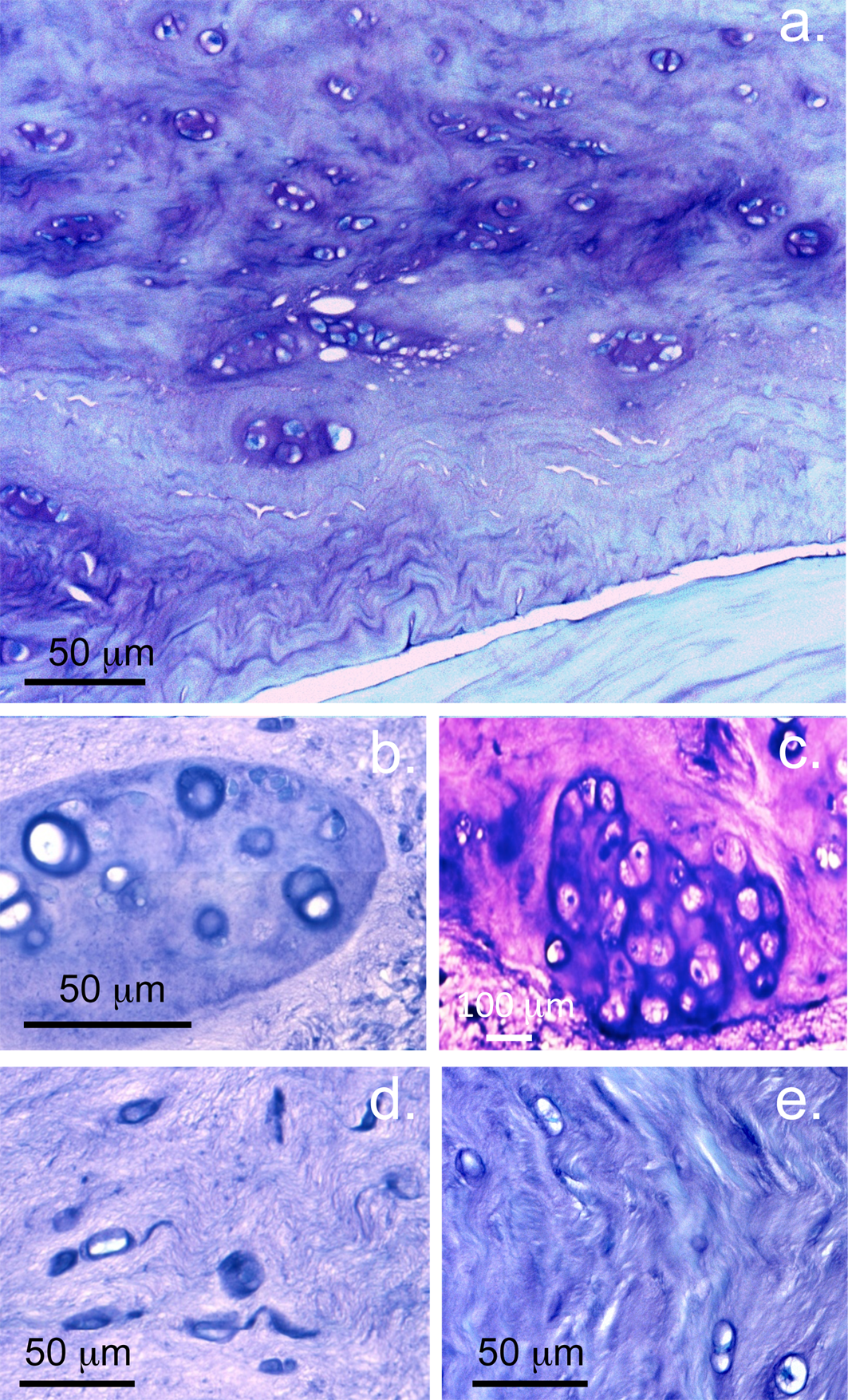

Supplement: Supplementary file 8 — Figure S8. Toluidine blue stained IVD tissue sections depicting cell clusters/cloning, chondroid cell nests and normal NP cell morphology. Examples of cell clustering associated with an annular lesion (A) and chondroid cell nests in the NP of a normal IVD (B, C) compared to cells in the central NP (D) and its margins with the AF (E). Occasional NP cells appear as doublets but are dissimilar to the chondroid cell nests contained in dense basophilic sacs which appear as larger groups of cells. These are similar to the cell clusters seen associated with annular lesions in degenerate IVDs however the chondroid cell nests are not associated with a fibrillar ECM like in A, D, E. [file JSP2-1-e1037-s008.tif]
