# Supplementary material for: Efficacy of administered mesenchymal stem cells in the initiation and co‐ordination of repair processes by resident disc cells in an ovine (Ovis aries) large destabilizing lesion model of experimental disc degeneration
Source: JOR Spine. 2018 Oct 10;1(4):e1037. doi: 10.1002/jsp2.1037 (PMC6686814; doi:10.1002/jsp2.1037)
Supplement: Supplementary file 9 — Table S1. Histopathological scoring of ovine IVDs [file JSP2-1-e1037-s009.pdf]

Supplement Table 1

Supplement Table 1¶. Histopathological Scoring of Ovine IVDs
Abbreviations: IAF, inner AF; MAF, mid AF; OAF, outer AF; NP, nucleus pulposus; CEP, cartilaginous endplate.

Grade	Histopathological Features
**A. Toluidine blue/Fast Green Proteoglycan Staining**	
0	Fast green staining only of OAF, metachromatic Toluidine blue staining of IAF, intense metachromatic staining in NP, well defined CEP staining. Alternate AF lamellae discernable due to differing metachromatic staining intensities of adjacent lamellae
1	Moderately reduced metachromatic staining of MAF/IAF in vicinity of lesion, fast green staining of OAF only, normal metachromatic staining of NP and CEP
2	Reduced patchy metachromatic staining around lesion, fast green staining in OAF (no metachromasia)
3	Reduced metachromatic staining in NP compared to sham or NOC IVD, very faint or no metachromatic staining in OAF/MAF, fast green staining only in OAF.
**B. IVD Structure/Lesion Characteristics**	
0	Normal IVD structure with well defined annular lamellae, central NP and CEP
1	Lesion evident in MAF, normal NP morphology
2	Lesion evident in MAF/IAF, lesion but may not be apparent in OAF due to spontaneous repair, IAF lamellae may be inverted and have anomalous distortions in normal lamellar architecture.
3	Bifurcation/propagation of lesion from MAF/IAF into NP margins, mild delamellation, when more extensive may lead to concentric tears between lamellae in MAF/IAF
4	Propagation of lesion into NP, with disruption in normal NP structure, distortion of annular lamellae into atypical arrangements-severe delamellation, separation of translamellar cross bridges
5	Lesion reaching through NP into contralateral posterior AF with disruption in normal NP structure.
**C. Cellular morphology**	
0	Normal, sparse distribution of typical single AF and NP fibrochondrocytes
1	Small groups of rounded chondrocytic cells (2-4 cells/group) in vicinity of annular lesion in IAF, occasional cell division in resident inner AF and NP cells
2	Moderate increase in well defined groups of rounded dividing cells (4-8 per group) in vicinity of lesion and with penetrating blood vessels associated with the lesion site, well defined chondroid cell colonies in NP contained within a dense basophilic matrix with little fibrillar material evident around the cells contrasting with NP cells
3	Numerous cell clones around IAF/MAF lesion, chondroid cell nests in NP containing >50 cells
**D. Blood Vessel Ingrowth**	
0	Very occasional vessels in outermost annular lamellae, occasional capillaries in CEP
1	Occasional blood vessels in OAF and MAF
2	Moderate number of blood vessels in IAF
3	Extensive ingrowth of vessels in IAF/MAF and along lesion margins
**E. Influx of cells into the lesion site**	
0	Normal cell distribution in OAF, MAF, IAF and NP
1	Slight influx of cells mainly in outer AF
2	Moderate influx of cells throughout AF
3	Large influx of cells throughout AF
4	Heavy influx of cells throughout AF particularly in inner AF and around lesion
**F. Histopathological features not covered elsewhere**	
2	Chondroid metaplasia in AF
2	Cystic degeneration affecting ≥ 5% NP
3	Extensive cystic degeneration affecting ≥ 20% NP
4	"bare " fibrillar elements in NP due to loss of ground substance, confirmed by a paucity of toluidine blue metachromasia affecting ≥ 20% of NP and also evident as a reduced disc height

¶ Table reproduced from [52].
