# Supplementary material for: Efficacy of administered mesenchymal stem cells in the initiation and co‐ordination of repair processes by resident disc cells in an ovine (Ovis aries) large destabilizing lesion model of experimental disc degeneration
Source: JOR Spine. 2018 Oct 10;1(4):e1037. doi: 10.1002/jsp2.1037 (PMC6686814; doi:10.1002/jsp2.1037)
Supplement: Supplementary file 10 — Table S2. qRTPCR primer details [file JSP2-1-e1037-s010.pdf]

**Supplement Table 2**. qRTPCR primer details

| **Gene** | **Species/ Accession** | **Sequence 5'-3'** | **Tm** | **Product Size** |
|---|---|---|---|---|
| *COL1A1* | *Ovis aries*<br>AF129287 | F -ATCCCTGGACAACCTGGACTTC<br>R -TCATCATAGCCGTAAGACAACTGG | 57 | 107 |
| *COL2A1* | *Bos taurus*<br>X02420 | F – TGACCTGACGCCCATTCATC<br>R – TTTCCTGTCTCTGCCTTGACCC | 55 | 154 |
| *ACAN* | Bos tauris<br>U76615 | F – TCACCATCCCCTGCTACTTCATC<br>R – TCTCCTTGGAAATGCGGCTC | 58 | 105 |
| *IL1RN* | *Ovis aries*<br>XM004005903.11 | F – TTCGCCTTCATCCGCTCTGACAAC<br>R-CTGCTGTAAGTAGAACCTGGTGACC | 59 | 159 |
| *MMP2* | *Ovis Aris*<br>AF267159 | F – TGCTACCACCTCCAACTACGATG<br>R – GTGCCAGTATCAATGTCAGGGG | 60 | 240 |
| *MMP3* | *Bos taurus*<br>AF135232 | F – TCCCCCAGTTTCCCCTAATG<br>R – GATTTCTCCCCTCAGTGTGCTG | 58 | 124 |
| *MMP9* | *Bos taurus*<br>X78324 | F -AGGTGAATCAGGTGGACTATGTGG<br>R -AGAAAGGAAGGTGGGAAGAGAGG | 59 | 221 |
| *MMP13* | *Ovis aries*<br>AY091604 | F – TGACAGGCAGACTTGATGATAAC<br>R – CATTTTGGACCACTTGAGAGTTC | 58 | 113 |
| *ADAMTS4* | *Bos taurus*<br>NM181667 | F – AACTCGAAGCAATGCACTGGT<br>R – TGCCCGAAGCCATTGTCTA | 60 | 149 |
| *ADAMTS5* | *Bos taurus*<br>AF192771 | F – GCATTGACGCATCCAAACCC<br>R-CGTGGTAGGTCCAGCAAACAGTTAC | 55 | 97 |
| *TIMP1* | *Ovis aries*<br>S67450 | F – GGTTCAGTGCCTTGAGAGATGC<br>R –GGGATAGATGAGCAGGGAAACAC | 57 | 265 |
| *TIMP3* | *Bos taurus*<br>NM174473 | F – CTTCCTTTGCCCTTCTCTACCC<br>R – TCTGGTCAACCCAAGCATCG | 57 | 286 |
